# Spontaneous Isopeptide Bond Formation as a Powerful Tool for Engineering Site-Specific Antibody-Drug Conjugates

**DOI:** 10.1038/srep39291

**Published:** 2016-12-16

**Authors:** Vanessa Siegmund, Birgit Piater, Bijan Zakeri, Thomas Eichhorn, Frank Fischer, Carl Deutsch, Stefan Becker, Lars Toleikis, Björn Hock, Ulrich A. K. Betz, Harald Kolmar

**Affiliations:** 1 Institute of Organic Chemistry and Biochemistry, Technische Universität Darmstadt, 64287 Darmstadt, Germany; 2 Merck KGaA, Frankfurter Straße 250, 64293 Darmstadt, Germany; 3 EMD Serono Research & Development Institute, Inc., 45A Middlesex Turnpike, Billerica, MA 01821, USA

## Abstract

Spontaneous isopeptide bond formation, a stabilizing posttranslational modification that can be found in gram-positive bacterial cell surface proteins, has previously been used to develop a peptide-peptide ligation technology that enables the polymerization of tagged-proteins catalyzed by SpyLigase. Here we adapted this technology to establish a novel modular antibody labeling approach which is based on isopeptide bond formation between two recognition peptides, SpyTag and KTag. Our labeling strategy allows the attachment of a reporting cargo of interest to an antibody scaffold by fusing it chemically to KTag, available via semi-automated solid-phase peptide synthesis (SPPS), while equipping the antibody with SpyTag. This strategy was successfully used to engineer site-specific antibody-drug conjugates (ADCs) that exhibit cytotoxicities in the subnanomolar range. Our approach may lead to a new class of antibody conjugates based on peptide-tags that have minimal effects on protein structure and function, thus expanding the toolbox of site-specific antibody conjugation.

The conjugation of small molecule drugs to antibodies represents a promising strategy for the development of cancer therapeutics. By harnessing the capacity of antibodies to home in on specific targets and combining that with the cytotoxic capability of small molecule drugs, antibody-drug conjugates (ADCs) can be generated to deliver lethal payloads to cancer cells with precision, while minimizing the off-target effects of cytotoxic drugs to increase the therapeutic index^1^.

First generation ADCs employing statistic conjugation of cytotoxic payloads via reduced cysteines or lysines led to heterogenous populations with limited therapeutic index suffering from a low efficacy and inconsistent *in vivo* performance^2^,^3^. Initial attempts to generate homogenous ADCs with a defined stoichiometry relied on the mutation of selected interchain cysteines to serines and conjugation of the cytotoxic payload to the remaining accessible cysteines originating from reduction^4^. Since then, several elegant methods have been developed to conjugate drugs to antibodies in a site-specific manner^5^,^6^. Chemical methods include the site-specific chemical conjugation through engineered cysteines^7^ or selenocysteines^8^,^9^, cysteine containing tag with perfluoroaromatic reagents^10^ and conjugation to reduced intermolecular disulfides by re-bridging dibromomalemides^11^, bis-sulfone reagents^12^, and dibromopyridazinediones^13^. In addition, several enzymatic and chemoenzymatic conjugation approaches have been reported including the use of engineered galactosyl- and sialyltransferases^14^, formyl glycine generating enzyme (FGE)^15^, phosphopantetheinyl transferases (PPTases)^16^, sortase A^17^, and microbial transglutaminase^18^,^19^,^20^, an enzyme forming an isopeptide bond between a glutamine side-chain and an amine-donor substrate.

Here we sought to explore a new method for engineering ADCs. Spontaneously forming intramolecular isopeptide bonds—peptide bonds that form outside of the protein main chain—were first discovered a decade ago and were found to provide remarkable stability to outer-membrane proteins of Gram-positive bacteria^21^. Using these protein scaffolds, Zakeri *et al*. engineered a series of genetically programmable peptide-protein partners that are able to spontaneously reconstitute via covalent and irreversible isopeptide bond formation^22^,^23^. One of these peptide-protein partners was obtained by engineering and splitting the *C*-terminal beta-strand of the CnaB2 domain from the fibronectin adhesion protein FbaB of *Streptococcus pyogenes* that is able to reconstitute with the protein by forming an intramolecular isopeptide bond between an aspartate and a lysine residue catalyzed by an opposed glutamate^22^. Further engineering of the CnaB2 domain and splitting it into three parts generated the synthetic enzyme SpyLigase that is able to direct the formation of an isopeptide bond between the two peptides SpyTag and KTag (Fig. 1A)^24^. Since the tags can be genetically fused to various proteins, these protein superglues have emerged as useful tools to covalently and specifically assemble linear and branched protein structures, thereby enabling the generation of new protein architectures via modular assembly^25^.

## Results

### Expression of peptide-tagged IgG1 antibody Fc domains, SpyLigase and synthesis of labeled peptides

**Figure 1:**
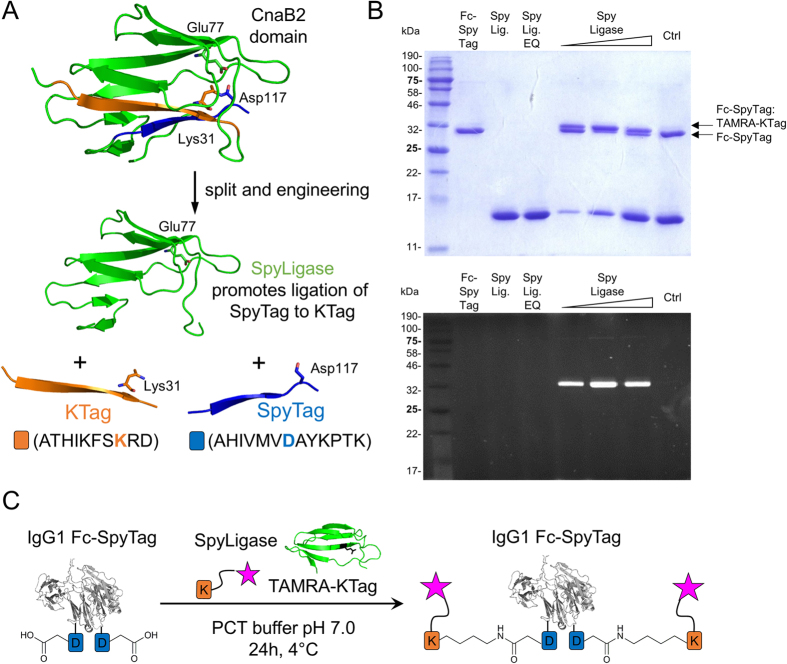
Site-specific conjugation of Spy-tagged IgG1-Fc with 5/6-carboxytetramethylrhodamine (TAMRA)-KTag by SpyLigase-mediated isopeptide bond formation. (**A**) Cartoon illustrating the splitting strategy to form SpyLigase from the CnaB2 domain. The two peptide tags, KTag (orange) with the reactive lysine and SpyTag (blue) with the reactive aspartic acid, can be ligated by the remaining protein domain (SpyLigase, green) by isopeptide bond formation. Active-site residues involved in the reaction are indicated (PDB 2X5P). (**B**) SDS-PAGE, Coomassie staining (top), and in-gel fluorescence (bottom) of the reduced Fc-fluorophore conjugates. Reactions were conducted with increasing concentration of SpyLigase (1, 3, and 10 mol eq. over Fc) and 10-fold excess of TAMRA-KTag. Control reactions (Ctrl) were performed by using 10 mol eq. of SpyLigase EQ. (**C**) Scheme of SpyLigase-mediated IgG1-Fc-SpyTag labeling using TAMRA-KTag.

To test whether the SpyLigase-catalyzed peptide ligation approach could also be applied for the covalent attachment of small reporting molecules such as fluorescent dyes, biotin or cytotoxins to antibody scaffolds, we fused SpyTag (13aa) and KTag (10aa) genetically to the *C*-terminus of an Fc domain from an IgG1 antibody via a short GS-linker. In order to facilitate analysis of conjugates, an N297A mutation was introduced into the IgG1-Fc gene by site-directed mutagenesis to remove the natural glycosylation site. The peptide-tagged antibody fragments were transiently expressed in HEK293F cells and subsequently purified by protein A affinity chromatography. SpyTag and KTag-peptides with adjacent *N*-terminal GSG-spacer were synthesized on solid support by semi-automated SPPS using a Rink amide (RAM) resin yielding peptide carboxamides after cleavage. KTag was further extended with a GY-dipeptide at the carboxy terminus as described^24^. 5/6-Carboxytetramethylrhodamine (TAMRA), a fluorophore commonly used for the preparation of protein conjugates, and biotin were coupled to the resin-bound peptide amino terminus via standard amide coupling chemistry using 2-(1H-benzotriazol-1-yl)-1,1,3,3-tetramethyluronium hexafluorophosphate (HBTU) and N,N-Diisopropylethylamine (DIPEA). After cleavage from the resin, labeled peptides were purified by semi-preparative RP-HPLC and analyzed by ESI-MS. SpyLigase and its inactive mutant SpyLigase EQ were obtained in yields of 20 mg per liter by expression in *Escherichia coli* and purification by affinity chromatography followed by size-exclusion chromatography (SEC)^24^. The purity of the proteins was >95% as determined by denaturing SDS-PAGE gel electrophoresis (Fig. 1B, upper panel, and S1, lane 2–4).

### Conjugation of labeled peptides to antibody Fc domains by SpyLigase-mediated isopeptide bond formation

We next performed conjugation reactions of labeled-peptides to the peptide-tagged Fc domains at pH 7.0 in the presence of the protein stabilizer trimethylamine N-oxide (TMAO) for 18–24 h at 4 °C. Each reaction contained peptide-tagged IgG1-Fc, labeled peptide counterpart (20 eq.), and SpyLigase (1–10 eq.). All possible combinations of SpyTag and KTag with the respective TAMRA or biotin labeled counterpart were assayed. Covalent ligation reactions were analyzed by boiling the samples for 5 min in SDS-loading buffer and subsequent SDS-PAGE analysis. Reaction products were visualized by Coomassie staining, fluorescence readout or western blotting (not shown). For all combinations the formation of a new product, stable to boiling in SDS, with a slightly slower migration behavior consistent with isopeptide bond formation between SpyTag and KTag was observed (Fig. 1B, upper panel, and S1, lane 5–7). This product was not observed when SpyLigase was replaced by its inactive Glu77 to Gln mutant (SpyLigase EQ). This mutant was not able to catalyze the spontaneous isopeptide bond formation due to the lack of the required glutamate residue (Fig. 1B, upper panel, and S1, lane 8). These results suggested that SpyLigase covalently links KTag and SpyTag in a site-specific manner. The conjugation efficiencies for Fc-SpyTag with TAMRA-KTag were estimated to be 40–60%. Here, reactions with three equivalents of SpyLigase seemed to be most efficient (Fig. 1B, upper panel, lane 6). Conjugation of the Fc-KTag with TAMRA-SpyTag was observed to be less efficient, which may be caused by a shorter GS-linker that was used to fuse KTag to the Fc (Figure S1 lane 5–7).

### SpyLigase-mediated conjugation of labeled peptides to Spy-tagged antibodies

Having proven our site-specific labeling approach on antibody domains, we extended the application for the preparation of defined antibody conjugates of the anti-EGFR monoclonal antibody cetuximab (Erbitux^®^). The cetuximab fusion proteins with SpyTag at the *C*-terminus of the heavy chains, the light chains, and at both chains were obtained by expression in HEK293F cells and purified by protein A affinity chromatography. The yields were comparable to those obtained for the unmodified antibody (40–60 mg per liter cell culture). SEC analysis revealed no significant aggregation behavior of the peptide-tagged antibodies (Figure S2). Antibodies were then conjugated to TAMRA-KTag using SpyLigase under previously determined reaction conditions. Reaction products were analyzed by in-gel fluorescence and Coomassie staining following SDS-PAGE gel electrophoresis. Fluorescent bands corresponding to the conjugated antibody’s heavy and light chains and a mobility shift of the conjugated light chain confirmed that SpyLigase-promoted site-specific labeling can also be applied on full-length IgG1-antibodies (Figure S3, lane 2–4). These results indicated a chemically highly specific conjugation reaction due to the required assembling of SpyTag/KTag and SpyLigase to form an active complex mediating isopeptide bond formation.

### Synthesis of cytotoxin-peptide payloads and conjugation to Spy-tagged antibodies

**Figure 2:**
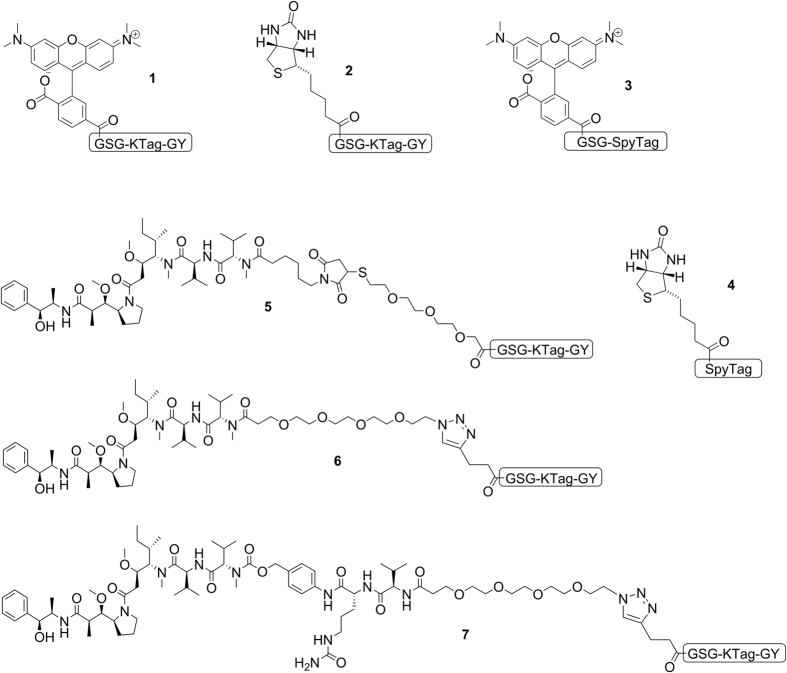
Overview of different cargoes of interest coupled to the *N*-terminus of KTag or SpyTag used in this study: (**1**) TAMRA-KTag, (**2**) Biotin-KTag, (**3**) TAMRA-SpyTag, (**4**) Biotin-SpyTag, (**5**) MMAE-nc-KTag (amide bond), (**6**) MMAE-nc-KTag (click chemistry), (**7**) MMAE-vc-KTag (click chemistry). Payloads **1**–**4** were subjected to SpyLigase-mediated IgG1-Fc conjugation whereas payloads **1** and **5**–**7** were used for conjugation to the antibody cetuximab as SpyTag fusion.

Next, we wanted to investigate whether SpyLigase-catalyzed conjugation could also be applied for the coupling of drug molecules to antibodies to generate site-specific ADCs. For this purpose, we designed and synthesized a set of Monomethyl auristatin E (MMAE)-peptide payloads differing in the nature of the used linker and the chemistry used for peptide-coupling (Fig. 2, compound **5**–**7**). MMAE was designed with either a non-cleavable (nc) maleimide-thiol linker (compound **5**), without linker (compound **6**), or with a cleavable valine-citrulline-*p*-aminobenzylcarbamate (vc-PABC) linker^7^,^26^,^27^ (compound **7**). In general, all payloads contained polyethylene glycol (PEG) spacer-units to increase the overall toxin solubility and to increase the toxin-peptide distance. To persue two different chemical strategies, toxins were either provided with an azide functionality for copper(I)-catalyzed azide-alkyne cycloaddition (CuAAC) or with an carboxyl group for amide coupling. For peptide-toxin coupling in solution by CuAAC, resin-bound KTag was first coupled to propargylacetic acid to obtain an *N*-terminal alkyne moiety before it was cleaved from the resin and purified. The carboxy functionalized MMAE was coupled directly to the *N*-terminus of the resin-bound peptide, requiring an acidic cleavage step of the entire MMAE-payload. As a proof of principle, cetuximab fused to SpyTag at the heavy chains was used for conjugation. These reactions were carried out in phosphate-citrate buffer pH 7.0 in the presence of TMAO for 18–24 h at 4 °C. Each reaction contained Spy-tagged antibody, 15 eq. of the toxic payload, and 3 eq. of SpyLigase. Reactions were analyzed by hydrophobic interaction chromatography (HIC) and drug-to-antibody ratios (DAR) were determined. Using payload **5**, the highest observable DAR was only 0.57 (Figure S4). With the payloads from peptide-coupling via click-chemistry, formation of about 80% of the desired species with two drug molecules was obtained, resulting in a DAR of 1.76 for payload **5** and a DAR of 1.66 for **7**. Control reactions with either SpyLigase EQ, without SpyLigase, and without payload did not result in the formation of a new product. This was analyzed by HIC, confirming also the specificity of the reaction (Figure S5).

### MS analysis of Spy-tagged ADCs

Next, we confirmed the identity of the peptide-tagged ADCs and the specificity of the conjugation reaction by mass spectrometry. Intact mass analysis of ADCs by MALDI-MS indicated the attachment of two payload molecules per antibody by a mass shift of 5252 Da (calc. 5349 Da) for payload **6** and 6202 Da (calc. 6159 Da) for payload **7** (Figure S7) compared to unmodified Spy-tagged cetuximab. This observation was verified by mass analysis of reduced ADCs showing a mass shift of 2735 Da (calc. 2675 Da) and 3116 Da (calc. 3080 Da) for payload **6** and **7**, respectively (Figure S8). In order to confirm the identity of the ADCs, a more accurate mass determination was obtained by LC-ESI-MS showing a mass shift of 2677 Da for compound **6** (calc. 2675 Da) (Figure S9). Tryptic digestion of the ADCs resulted in a peptide fragment composed of amino acids derived from both SpyTag and KTag which are connected to each other via an isopeptide bond between Asp and Lys residues (Figure S13). This fragment was successfully identified in both ADC samples but not in the unconjugated antibody control. Further identification of the peptide was conducted by sequencing using Tandem-MS (Figures S14–S16). Unmodified SpyTag fragment, however, was only observed in minor amounts confirming the efficiency of our approach (Figure S17). No unspecific conjugation of the antibody light chain was observed (Figure S18).

### *In vitro* cytotoxicity of Spy-tagged ADCs

**Figure 3:**
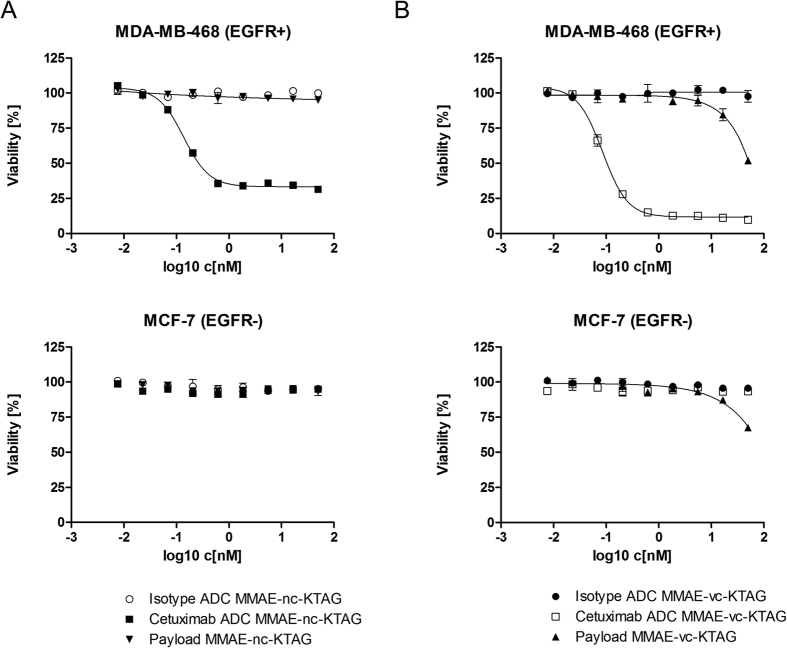
Cytotoxicity induced by Spy-tagged cetuximab ADCs using EGFR overexpressing MDA-MB-468 and EGFR-negative MCF-7 breast cancer cell lines. (**A**) *In vitro* cell killing of ADCs with non-cleavable (nc) MMAE payload **6** (■). (**B**) *In vitro* cell killing of ADCs with cleavable (vc) MMAE payload **7** (□). Incubation times were 3 days. Trastuzumab ADCs were used as isotype controls (○ and ●). Compound **6** (▼) and compound **7** (▲) were also assayed as controls. IC_50_ values were calculated as mean values from two independent experiments.

To determine the cell killing activities of the Spy-tagged cetuximab-based ADCs *in vitro*, ligation reactions were scaled up and ADCs were purified by protein A chromatography in order to remove excess payload and SpyLigase. *In vitro* cytotoxicity was determined using two different breast cancer cell lines, MDA-MB-468 and MCF-7, the former one expressing high levels of EGFR (epidermal grow factor receptor, EGFR+), the latter one without EGFR expression (EGFR-)^28^. ADCs with payloads that were synthesized by CuAAC with either non-cleavable linker (compound **6**) or with cleavable linker (compound **7**) were compared to each other to investigate an effect of the different linkers on efficacy and potency. The ADCs were tested in a serial dilution with the highest concentration of 50 nM for three days. As expected, cetuximab-based ADCs effect on MDA-MB-468 cells in a dose dependent manner (Fig. 3, upper panels). For the ADC with a non-cleavable linker the half-maximal cytotoxic effect was in the subnanomolar range (IC_50_ = 0.2 nM) (Fig. 3A, ■). In contrast the ADC with a cleavable linker showed an even higher *in vitro* potency (IC_50_ = 0.1 nM) (Fig. 3B, □) which was probably due to the cathepsin B-mediated cleavage of the linker in the lysosome releasing the free toxin upon cellular uptake. No effect on cell viability was observed with the same ADCs on the EGFR-negative cell line MCF-7 (Figure 3, lower panels). To confirm the receptor-mediated internalization and cell killing of the anti-EGFR ADCs, anti HER2 (human epidermal grow factor receptor 2) Spy-tagged trastuzumab ADCs were prepared with the same payloads under identical reaction conditions and tested as isotype controls. The free payload was also used as control. MDA-MB-468 cells do not express HER2 whereas MCF-7 cells exhibit a low expression of HER2^28^. The cell viability of both cell lines was not affected by the isotype control ADCs (Fig. 3, ○ and ●), demonstrating that the anti-EGFR ADCs generated by using the catalytic activity of SpyLigase selectively kill EGFR-overexpressing cells in a receptor-dependent manner. A cell killing effect of free payload **7** was observed at the highest concentration of 50 nM (Fig. 3B, ▲).

## Conclusion

In conclusion, we have developed a modular bioconjugation approach based on isopeptide bond formation between two peptide tags catalyzed by SpyLigase. This approach was used as a tool for the site-specific conjugation of small reporting molecules like fluorescent TAMRA and biotin to antibodies and for the generation of homogenous ADCs. ADCs prepared by fusing SpyTag to the *C*-terminus of the anti-EGFR monoclonal antibody cetuximab and attaching a cytotoxic compound to the chemically synthesized KTag were characterized regarding their biophysical and cytotoxic properties *in vitro*. The obtained ADCs performed specifically and were observed to exhibit subnanomolar IC_50_ values. We assume that this technology can also be used for engineering ADCs with a higher payload density since it has been described that the used peptide tags were also reactive when placed at internal sites of a protein^24^. The feasibility of such a peptide-tagged ADC approach has recently been proven by the introduction of small substrate sequences for enzyme-promoted conjugation at different constant region loops positions of trastuzumab for efficient site-specific ADC preparation^16^. The possibility to place the peptide-tags at multiple sites within the antibody scaffold would clearly provide an advantage compared to covalent peptide labeling via sortases that exclusively allow for *C*-terminal payload conjugation^17^,^29^,^30^,^31^. In addition to microbial transglutaminase our approach offers the opportunity for site-specific and efficient antibody conjugation based on a stable isopeptide bond.

## Methods

### Expression and purification of SpyLigase

The plasmid coding for SpyLigase (pDEST14-SpyLigase) was purchased from Addgene (Addgene ID 51722). The plasmid coding for the inactive form of SpyLigase (pDEST14-SpyLigase EQ) was generated by Quick Change Side-Directed Mutagenesis by introducing an E77Q mutation according to the literature^22^,^24^. SpyLigase expression in *E. coli* BL21 DE3 pLysS (Stratagene) and purification via immobilized metal ion affinity chromatography (IMAC) was conducted according to the literature^22^,^24^. After dialyzing proteins in a 1,000-fold excess of PBS overnight at 4 °C, size-exclusion chromatography (SEC) was performed using a HiPrep (16/60) Sephacryl S-200 HR column (GE Healthcare) and PBS. Proteins were concentrated by centrifugation dialysis using Amicon Ultra-15 (Merck Millipore, NMWL 3000 Da) and protein concentrations determined at 280 nm using a nano spectrophotometer (MW = 11474.3 g/mol, ε_280_ = 15930 M^−1^m^−1^). A protein yield of about 20 mg per liter culture could be obtained. Proteins were supplemented with 10% (v/v) glycerol, aliquots frozen in liquid nitrogen, and stored at −80 °C.

### Expression and purification of IgG1 Fc and antibodies fused to peptide tags

IgG1 Fc (pEXPR-IgG1 Fc) was fused to either SpyTag (AHIVMVDAYKPTK) or KTag (ATHIKFSKRD) with additional GY-dipeptide at the *C*-terminus by using standard molecular cloning techniques. Native IgG1 glycosylation at position N297 was eliminated by introducing a N297A mutation by Quick Change Side-Directed Mutagenesis. Plasmids coding for chimeric cetuximab (C225, Erbitux^®^) and trastuzumab (Herceptin^®^) were kindly provided by Merck KGaA (Darmstadt). Cetuximab and trastuzumab variants containing SpyTag at the *C*-terminus of either the heavy chains, the light chains, or at both chains were prepared by standard molecular cloning techniques. A (GSG)_2_-linker was introduced between the *C*-terminus of the antibody and SpyTag. IgG1 Fc and full-length antibodies were transiently expressed from HEK293F cells using the Expi293 Expression System (Life Technologies). Supernatants containing secreted proteins were conditioned and applied to spin columns with PROSEP-A Media (Montage, Merck Millipore). Columns were washed with 1.5 M Glycine/NaOH, 3 M NaCl, pH 9.0 and proteins eluted with 0.2 M Glycine/HCl pH 2.5 into 1 M Tris/HCl pH 9.0. Eluted proteins were dialyzed in 1 × DPBS (Life Technologies) using Amicon Ultra-15 (Merck Millipore, NMWL 10000 Da) and stored at 4 °C.

### Conjugation of peptide-tagged IgG1 Fc

Antibody fragments (IgG1 Fc) fused to either SpyTag or KTag at the *C*-terminus were mixed at 5 μM with 50 μM of TAMRA-KTag or TAMRA-SpyTag, respectively, and incubated in the presence of 5–50 μM of SpyLigase (1–10 mol eq.) for 24 h in 40 mM Na_2_HPO_4_, 20 mM citric acid buffer, pH 5.0, with addition of 1.5 M trimethylamine N-oxide (Sigma-Aldrich) to give a final pH of 7.0 (PCT buffer) at 4 °C. Control reactions using 50 μM of an inactive SpyLigase (EQ, E77Q) were performed simultaneously. Reactions were stopped by the addition of SDS loading buffer and samples heated at 95 °C for 5 min. SDS-PAGE was performed on 15% polyacrylamide gels at 40 mA for approximately 45 min. Gels were analyzed by fluorescence readout using a Versa Doc Imaging System 5000 (Bio-Rad) and afterwards stained with Coomassie Blue.

### SpyLigase-mediated antibody conjugation

Cetuximab or trastuzumab fused to SpyTag at the *C*-terminus of the heavy chain, light chain, or at both chains was mixed at 6 μM with 120 μM of TAMRA-KTag and incubated in the presence of 18 μM of SpyLigase for 24 h in 40 mM Na_2_HPO_4_, 20 mM citric acid buffer, pH 5.0, with addition of 1.5 M trimethylamine N-oxide (Sigma-Aldrich) to give a final pH of 7.0 at 4 °C (PCT buffer). Antibodies conjugated to TAMRA-KTag were analyzed by SDS-PAGE on 15% polyacrylamide gels and fluorescently labeled heavy and/or light chains visualized by fluorescence readout using a Versa Doc Imaging System 5000 (Bio-Rad). For toxin conjugations, cetuximab or trastuzumab fused to SpyTag at the *C*-terminus of the heavy chain was incubated at 6 μM with 90 μM of the respective MMAE-KTag payload and incubated in the presence of 18 μM of SpyLigase under the above described reaction conditions. Antibody drug conjugates were evaluated by hydrophobic interaction chromatography (HIC) on a TSKgel Butyl-NPR column (Tosoh Bioscience, 4.6 mm x 3.5 cm, 2.5 μm) using an Agilent Infinity 1260 HPLC. The HIC method was applied using a mobile phase of 1.5 M (NH_4_)_2_SO_2_, 25 mM Tris-HCl pH 7.5 (Buffer A) and 25 mM Tris-HCl pH 7.5 (Buffer B). ADCs (45 μg) in 0.75 M (NH_4_)_2_SO_2_ were loaded and eluted with a gradient consisting of 2.5 min 0% Buffer B followed by a linear gradient into 100% Buffer B over 35 min with a flow rate of 0.9 ml/min. For preparative ADC preparations, reactions were scaled up and ADCs purified by protein A magnetic beads (Promega) according to the manufacturers protocol. Excess payload and SpyLigase were removed by rigorous washing with PBS before eluting ADCs with 100 mM citrate buffer pH 3.0 into 1 M Tris-HCl pH 9.0 neutralization buffer. ADCs were buffered into PBS by using PD MiniTrap G-25 colums and concentrated by centrifugation dialysis (100 K Amicon Ultra 0.5 Centrifugal Filters).

### *In vitro* cytotoxicity of peptide-tagged ADCs

*In vitro* cytotoxicity of peptide-tagged anti EGFR ADCs was determined by using MDA-MB-468 and MCF-7 breast cancer cells. MDA-MB-468 cells have a high EGFR expression level, but no HER2 expression^28^. MCF-7 cells show no EGFR expression, but a weak HER2 expression^28^. Cells were cultivated in appropriate culture media according to the manufacturer protocol. Cells were detached by trypsination, diluted and allowed to settle down in 96-well plates at a seeding density 12.5 × 10^4^ cells/ml (0.08 ml/well) for 24 h at 37 °C (5% CO_2_). ADCs and free payloads were diluted in appropriate culture media and added to the cells in various concentrations (ranging from 50 nM to 7.6 pM) following a three-day incubation at 37 °C (5% CO_2_). All concentrations were assayed in duplicates. Cell proliferation was determined by adding CellTiter-Glo (Promega) and luminescence readout (Synergy 5, BioTek).

### General for peptide chemistry

All chemicals and solvents for peptide synthesis, analysis, and isolation were purchased from Iris Biotech, Agilent Technologies, Sigma-Aldrich, or Roth and used without any further purification. MMAE-linker payloads were obtained from Syngene.

### Peptide solid-phase synthesis

Peptides were synthesized at a 0.1 mmol scale on an AmphiSpheres 40 RAM resin (Agilent, 0.37 mmol/g) by microwave-assisted Fmoc-SPPS using a Liberty Blue^TM^ Microwave Peptide Synthesizer. Activation of the respective carboxyfunctional amino acid (0.2 M) was performed by 1 M Oxyma/0.5 M *N,N*′-Diisopropylcarbodiimide (DIC). Deprotection of the aminoterminal Fmoc-group was achieved using 20% piperidine in DMF in the presence of 0.1 M Oxyma. During the synthesis cycles all amino acids were heated to 90 °C (histidine to 50 °C). Peptides were cleaved from the resin for analytical purposes by a standard cleavage cocktail of 94% TFA, 2% triethylsilane, 2% anisole, 2% H_2_O. Methionine containing peptide **2** was cleaved in the presence of dithiotreitol (DTT) to prevent oxidation. After 1 h of cleavage, peptides were precipitated in cold diethylether, washed with diethylether and dried *in vacuo* prior to LC-MS analysis.

### RP-HPLC

Peptides were analyzed by chromatography using an analytical RP-HPLC from Agilent Technolgies (920-LC or 1260 Infinity) using either a Phenomenex Hypersil 5 u BDS C18 LC column (150 × 4.6 mm, 5 μm, 130 Å) or a Phenomenex Luna 5 u C18 (2) column (150 × 4.6 mm, 5 μm, 100 Å). Peptides were isolated by semi-preparative RP-HPLC (Varian) using a Phenomenex Luna 5 u C18 LC column (250 × 12.2 mm, 5 μm, 100 Å). Eluent A (water) and eluent B (90% aq. MeCN) each contained 0.1% trifluoroacetic acid (TFA).

### ESI-MS analysis of peptides

ESI mass spectra were measured on a Shimadzu LCMS-2020 equipped with a Phenomenex Synergi 4 u Hydro-RP LC column (250 × 4.6 mm, 4 μm, 80 Å) by using an eluent system consisting of eluent A (0.1% aq. formic acid, LC-MS grade) and eluent B (100% acetonitrile containing 0.1% formic acid, LC-MS grade).

### MALDI-TOF-MS analysis of intact masses

20 μg of protein was used for non-reduced and reduced (30 min at 60 °C with 5 mM Dithiothreitol) sample preparation. Prior MALDI analysis on an Ultraflex III (Bruker, Bremen), samples were desalted and concentrated with C4 ZipTips (Millipore, Cork) corresponding to the manufactures guide. The prepared samples were directly mixed in equal volumes with saturated alpha-Cyano-4-hydroxy-cinnamic acid matrix (Bruker, Bremen) on a MTP AnchorChip 384 TF plate (Bruker, Bremen). The Ultraflex III was used in a linear positive mode and data acquisition was performed in a mass range between m/z 10000 to 180000. Per sample, 4000 laser shot were accumulated and finally smoothed and baseline subtracted.

### CapLC-ESI-MS analysis of intact masses

For intact mass analysis of heavy and light chain, 20 μg of each sample was mixed with 1% mercaptoethanol final concentration and reduced for 30 min. at 37 °C. Reduced samples were loaded directly onto a CapLC-MS system (1100 series, Agilent Technologies) coupled to a Synapt G1 HDMS (Waters, Milford, MA) operated in MS positive ion mode. 0.1% trifluoroacetic acid in water was used as solvent A and 0.1% trifluoroacetic acid in 70% n-propanol as solvent B. The samples were separated via a Zorbax SB300 C8 column (3.5 μm, 150 × 0.3 mm, Agilent Technologies), tempered at 75 °C. The gradient started with 3% B and a flow rate of 10 μL/min followed by a linear gradient from 2 to 39 min from 10% B to 60% B. Protein signals were recorded with a DA-detector at 214 and 280 nm. MS spectra were acquired from m/z 500 to 3000. Finally, intact protein mass was calculated applying the MaxEnt1 deconvolution algorithm.

### NanoLC-ESI-MSe analysis of tryptic peptides

Investigation of the payload conjugation site was performed by peptide mapping analysis using a slightly modified RapiGest SF Surfactant care and use protocol (Waters, Milford, MA). Briefly, 10 μg of each sample was mixed with RapiGest 0.1% final concentration and reduced for 30 min at 60 °C with 5 mM Dithiothreitol. Alkylation was performed with 15 mM iodoacetamide for 30 min at RT. Finally 1.5 μL of a 1 μg/μl trypsin solution were added and incubated overnight at 37 °C. After digestion, the sample was acidified by addition of 0.5% trifluoroacetic acid and analyzed by Nano-LC-MSe using a nanoAcquity UPLC coupled to a Synapt G1 HDMS (Waters, Milford, MA) operated in MSe mode. 0.1% formic acid in water was used as solvent A and 0.1% formic acid in acetonitrile as solvent B. Tryptic peptides were injected and trapped for 3 min on a Symmetry C18 pre-column (5 μm, 180 μm × 20 mm, Waters, Milford, MA) with a flow rate of 10 μl/min. Separation was performed using an UPLC 1.7 μm BEH130 column (C18, 75 μm × 100 mm, Waters) with a flow rate of 450 nl/min, starting with 2% B from 0 to 2 min followed by a linear gradient from 8–35% B for 67 min. The used MSe mode combines an alternating MS and MSe (full mass range fragmentation) function each second. Spectra were acquired from m/z 50 to 1600 and extracted ion chromatograms were analyzed manually.

### Synthesis of peptide conjugates 1–4

Resin-bound peptides were functionalized with 5/6-carboxytetramethylrhodamine (TAMRA, mixed isomers) or biotin at the free amino terminus by double-coupling using a preactivated carboxyl functionality. For activation, 2 eq. of TAMRA or biotin were incubated with 1.95 eq. of 2-(1H-benzotriazol-1-yl)-1,1,3,3-tetramethyluronium hexafluorophosphate (HBTU) in the presence of 4 eq. of *N*,*N*-Diisopropylethylamine (DIPEA) in DMF for 10 min shaking. Afterwards, the mixture was added to the resin-bound peptides (preswelled in 1:1 DMF/DCM) and coupling allowed to proceed for 2 h, respectively. Peptides were cleaved from the resin by using a standard cleavage cocktail of 94% TFA, 2% triethylsilane, 2% anisole, 2% H_2_O. Methionine containing SpyTag-peptides were cleaved in the presence of dithiotreitol (DTT) to prevent oxidation. After 2 h of cleavage, peptides were precipitated in cold diethylether, washed twice with diethylether, dried *in vacuo*, and afterwards purified by semi-preparative RP-HPLC.

### Synthesis of KTag with *N*-terminal alkyne functionality

KTag**-**peptide was equipped with an *N*-terminal alkyne functionality by coupling with propargylacetic acid (4-pentynoic acid) on resin. For this purpose, 4 eq. of the building block was preactivated for 10 min with 3.95 eq. of 2-(1H-benzotriazol-1-yl)-1,1,3,3-tetramethyluronium hexafluorophosphate (HBTU) and 8 eq. of *N*,*N*-Diisopropylethylamine (DIPEA) in DMF and afterwards added to the resin-bound peptide (preswelled in 1:1 DMF/DCM). Coupling was performed for 1.5 h twice and the resin washed subsequently with DMF, DCM, and diethylether for drying. The alkyne-functionalized peptide was cleaved from the resin using a standard cleavage cocktail of 94% TFA, 2% triethylsilane, 2% anisole, 2% H_2_O. After 2 h of cleavage, the peptide was precipitated in cold diethylether, washed twice with diethylether and dried *in vacuo*. The product was analyzed by LC-MS and purified by semi-preparative RP-HPLC.

### Synthesis of peptide-toxin conjugate 5

Resin-bound KTag-peptide was incubated with 1.2 eq. MMAE bearing a carboxy group in the presence of 1.14 eq. 2-(1H-benzotriazol-1-yl)-1,1,3,3-tetramethyluronium hexafluorophosphate (HBTU) and 2.4 eq. of *N*,*N*-Diisopropylethylamine (DIPEA) in DMF for 5 h at room temperature. Afterwards, the resin was washed twice with DMF, DCM, and diethylether. The toxin-functionalized peptide was cleaved from the resin using a cleavage cocktail of 85% TFA, 5% triethylsilane, 5% anisole, and 5% H_2_O for 1 h at room temperature. The peptide-toxin conjugate was precipitated in cold diethylether, washed twice with diethylether and dried *in vacuo*. Compound **5** was isolated by RP-HPLC.

### Synthesis of peptide-toxin conjugates 6 and 7

KTag was coupled to monomethyl auristatin E (MMAE) via Cu(I)-catalyzed azide-alkyne cycloaddition (CuAAC). For this purpose, the peptide bearing an amino terminal alkyne functionality (1 mg/ml) was reacted with 1.5 eq. MMAE holding an azide functionality in the presence of 4 eq. of Cu(II)-TBTA complex, 4 eq. of *N*,*N*-Diisopropylethylamine (DIPEA), and 4 eq. of sodium ascorbate for 5 h at room temperature in water. The water was degassed by flushing it with argon prior to usage. TBTA was dissolved at a concentration of 10 mM in 1:4 DMSO/tBuOH and the complex formed by addition of Cu(II)SO_4_ 5xH_2_O. The toxin was pre-dissolved in 50% MeCN. The reaction was monitored by HPLC-MS. Peptide-toxin conjugates **6** and **7** were isolated by semi-preparative RP-HPLC.

## Additional Information

**How to cite this article**: Siegmund, V. *et al*. Spontaneous Isopeptide Bond Formation as a Powerful Tool for Engineering Site-Specific Antibody-Drug Conjugates. *Sci. Rep.* **6**, 39291; doi: 10.1038/srep39291 (2016).

**Publisher's note:** Springer Nature remains neutral with regard to jurisdictional claims in published maps and institutional affiliations.

## Supplementary Material

Supplementary Information
