# Supplementary material for: Spontaneous Isopeptide Bond Formation as a Powerful Tool for Engineering Site-Specific Antibody-Drug Conjugates
Source: Sci Rep. 2016 Dec 16;6:39291. doi: 10.1038/srep39291 (PMC5159917; doi:10.1038/srep39291)
Supplement: Supplementary Information [file srep39291-s1.pdf]

Supporting Information

Spontaneous Isopeptide Bond Formation as a Powerful Tool for Engineering Site-Specific Antibody-Drug Conjugates

Vanessa Siegmund,[1,2] Birgit Piater,[2] Bijan Zakeri,[3] Thomas Eichhorn,[2] Frank Fischer,[2] Carl Deutsch,[2] Stefan Becker,[2] Lars Toleikis,[2] Björn Hock,[2] Ulrich A. K. Betz,[2] and Harald Kolmar[1*]

[1]Institute of Organic Chemistry and Biochemistry, Technische Universität Darmstadt, 64287 Darmstadt (Germany)

[2]Merck KGaA, Frankfurter Straße 250, 64293 Darmstadt (Germany)

[3]EMD Serono Research & Development Institute, Inc., 45A Middlesex Turnpike, Billerica, MA 01821 (USA)

Email: kolmar@Biochemie-TUD.de

Protein sequences

Purple indicates a 6x His Tag, glycine-serine (GS) linkers are underlined, green indicates a TEV-cleavage site, red indicates a mutation, blue indicates SpyTag (13aa), orange indicates KTag (10aa). IgG1 Fc constructs are preceded by a BM40 secretory signal sequence. Full-length antibody heavy and light chains are preceded by a 19-mer signal peptide (MKLPVRLLVLMFWIPASLS) to direct their secretion.

SpyLigase

MSYYHHHHHHDYDGQSGDGKELAGATMELRDSSGKTISTWISDGQVKDFYLYPGKYTFVET
AAPDGYEVATAITFTVNEQGQVTVNGKATKGGSGGSGGSGEDSATHI

SpyLigase EQ

MSYYHHHHHHDYDGQSGDGKELAGATMELRDSSGKTISTWISDGQVKDFYLYPGKYTFVQT
AAPDGYEVATAITFTVNEQGQVTVNGKATKGGSGGSGGSGEDSATHI

Fc-SpyTag

AENLYFQSGSEPKSCDKTHTCPPCPAPELLGGPSVFLFPPKPKDTLMISRTPEVTCVVVDVSHE
DPEVKFNWYVDGVEVHNAKTKPREEQYASTYRVVSVLTVLHQDWLNGKEYKCKVSNKALP
APIEKTISKAKGQPREPQVYTLPPSRDELTKNQVSLTCLVKGFYPSDIAVEWESNGQPENNYK
TTPPVLDSDGSFFLYSKLTVDKSRWQQGNVFSCSVMHEALHNHYTQKSLSLSPGGSGGSGAH
IVMVDAYKPTK

Native IgG1 glycosylation at N297 has been eliminated by introducing a N297A mutation.

Fc-KTag

AENLYFQSGSEPKSCDKTHTCPPCPAPELLGGPSVFLFPPKPKDTLMISRTPEVTCVVVDVSHE
DPEVKFNWYVDGVEVHNAKTKPREEQYASTYRVVSVLTVLHQDWLNGKEYKCKVSNKALP
APIEKTISKAKGQPREPQVYTLPPSRDELTKNQVSLTCLVKGFYPSDIAVEWESNGQPENNYK
TTPPVLDSDGSFFLYSKLTVDKSRWQQGNVFSCSVMHEALHNHYTQKSLSLSPGGSATHIKFS
KRDGY

Native IgG1 glycosylation at N297 has been eliminated by introducing a N297A mutation. An additional dipeptide GY was attached to the *C*-terminus of KTag.

C225-SpyTag-HC

QVQLKQSGPGLVQPSQSLSITCTVSGFSLTNYGVHWVRQSPGKGLEWLGVIWSGGNTDYNTP
FTSRLSINKDNSKSQVFFKMNSLQSNDTAIYYCARALTYYDYEFAYWGQGTLVTVSAASTKG
PSVFPLAPSSKSTSGGTAALGCLVKDYFPEPVTVSWNSGALTSGVHTFPAVLQSSGLYSLSSV
VTVPSSSLGTQTYICNVNHKPSNTKVDKRVEPKSCDKTHTCPPCPAPELLGGPSVFLFPPKPKD
TLMISRTPEVTCVVVDVSHEDPEVKFNWYVDGVEVHNAKTKPREEQYNSTYRVVSVLTVLH
QDWLNGKEYKCKVSNKALPAPIEKTISKAKGQPREPQVYTLPPSREEMTKNQVSLTCLVKGF
YPSDIAVEWESNGQPENNYKTTPPVLDSDGSFFLYSKLTVDKSRWQQGNVFSCSVMHEALHN
HYTQKSLSLSPGKGSGGSGAHIVMVDAYKPTK

C225-SpyTag-LC

DILLTQSPVILSVSPGERVSFSCRASQSIGTNIHWYQQRTNGSPRLLIKYASESISGIPSRFSGSGS
GTDFTLSINSVESEDIADYYCQQNNNWPTTFGAGTKLELKRTVAAPSVFIFPPSDEQLKSGTAS
VVCLLNNFYPREAKVQWKVDNALQSGNSQESVTEQDSKDSTYSLSSTLTLSKADYEKHKVY
ACEVTHQGLSSPVTKSFNRGECGSGGSGAHIVMVDAYKPTK

Trastuzumab-SpyTag-HC

EVQLVESGGGLVQPGGSLRLSCAASGFNIKDTYIHWVRQAPGKGLEWVARIYPTNGYTRYAD
SVKGRFTISADTSKNTAYLQMNSLRAEDTAVYYCSRWGGDGFYAMDYWGQGTLVTVSSAS
TKGPSVFPLAPSSKSTSGGTAALGCLVKDYFPEPVTVSWNSGALTSGVHTFPAVLQSSGLYSL
SSVVTVPSSSLGTQTYICNVNHKPSNTKVDKKVEPPKSCDKTHTCPPCPAPELLGGPSVFLFPP
KPKDTLMISRTPEVTCVVVDVSHEDPEVKFNWYVDGVEVHNAKTKPREEQYNSTYRVVSVL
TVLHQDWLNGKEYKCKVSNKALPAPIEKTISKAKGQPREPQVYTLPPSRDELTKNQVSLTCL
VKGFYPSDIAVEWESNGQPENNYKTTPPVLDSDGSFFLYSKLTVDKSRWQQGNVFSCSVMHE
ALHNHYTQKSLSLSPGGSGGSGAHIVMVDAYKPTK

Trastuzumab-LC

DIQMTQSPSSLSASVGDRVTITCRASQDVNTAVAWYQQKPGKAPKLLIYSASFLYSGVPSRFS
GSRSGTDFTLTISSLQPEDFATYYCQQHYTTPPTFGQGTKVEIKRTVAAPSVFIFPPSDEQLKSG
TASVVCLLNNFYPREAKVQWKVDNALQSGNSQESVTEQDSKDSTYSLSSTLTLSKADYEKHK
VYACEVTHQGLSSPVTKSFNRGEC

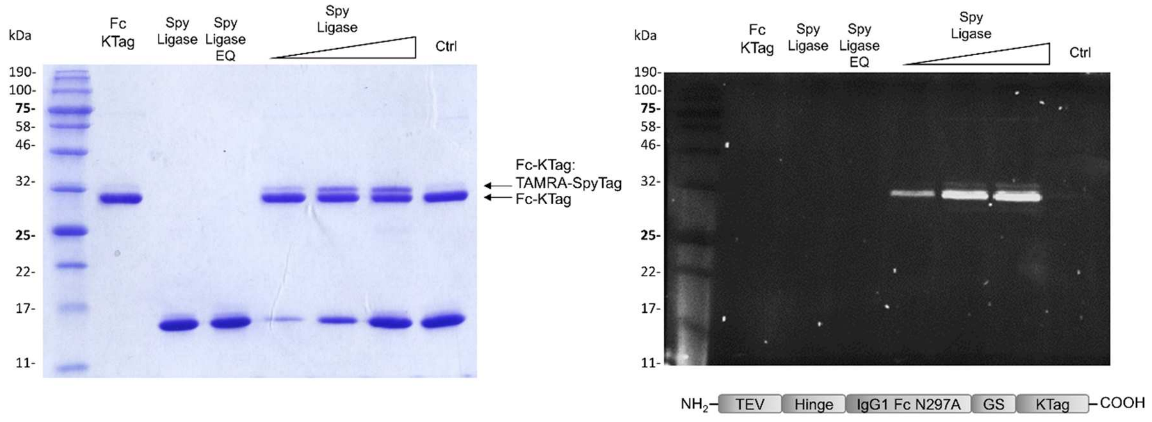

Figure S1. SpyLigase-mediated conjugation reactions of K-tagged IgG1 Fc with TAMRA-SpyTag. SDS-PAGE, Coomassie staining (left panel), and in-gel fluorescence (right panel) of the reduced Fc-fluorophore conjugates. Reactions were conducted with increasing concentrations of SpyLigase (1, 3, and 10 mol eq. over Fc) and 10-fold excess of TAMRA-SpyTag. Control reactions (Ctrl) were performed by using 10 mol eq. of SpyLigase EQ.

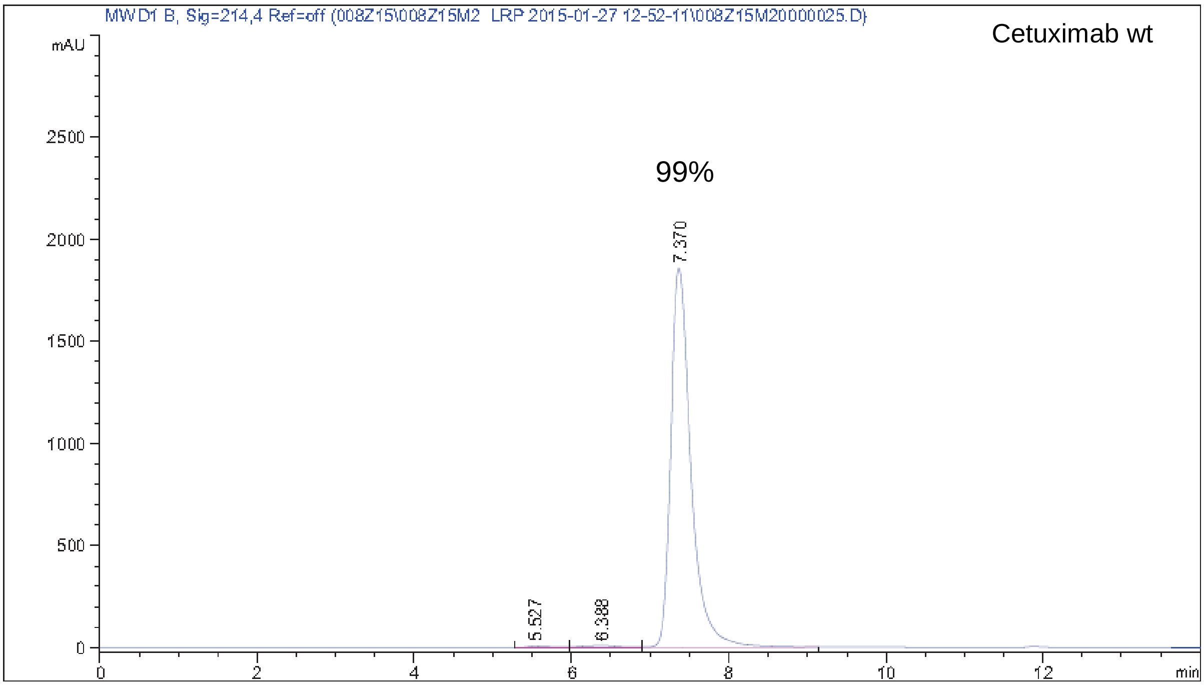
MWD1 B, Sig=214,4 Ref=off (008Z15\008Z15M2 LRP 2015-01-27 12-52-11\008Z15M20000025.D)
Cetuximab wt
99%
mAU
min
7.370
5.527
6.388

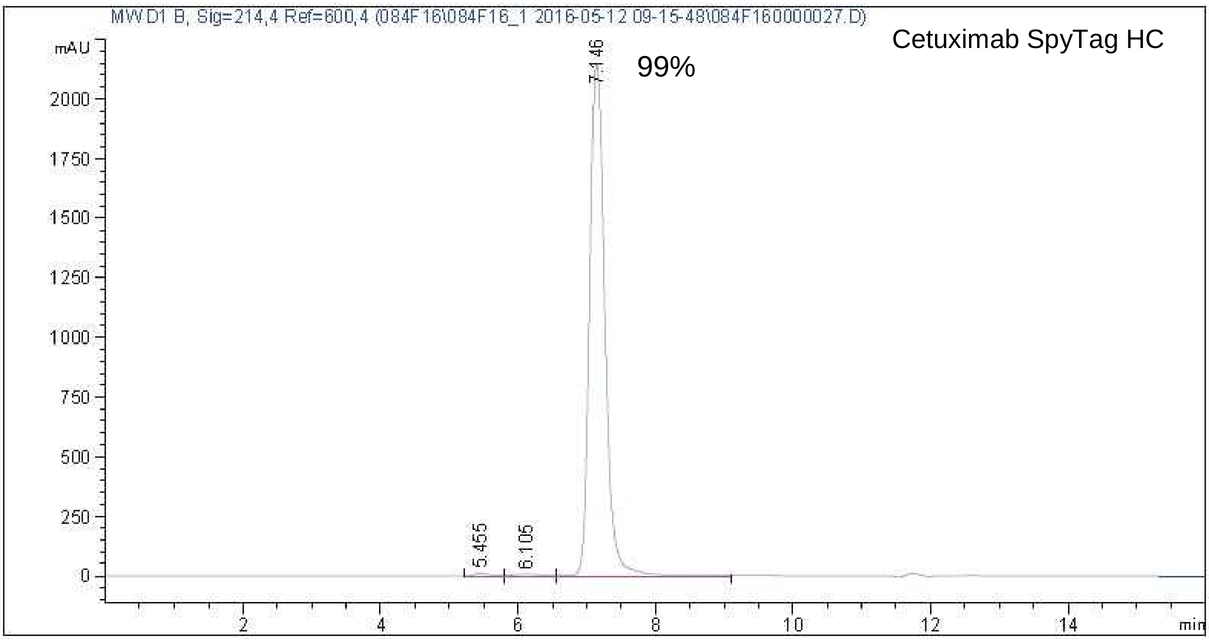
MWD1 B, Sig=214,4 Ref=600,4 (084F16\084F16_1 2016-05-12 09-15-48\084F160000027.D)
Cetuximab SpyTag HC
99%
mAU
min
7.146
5.455
6.105

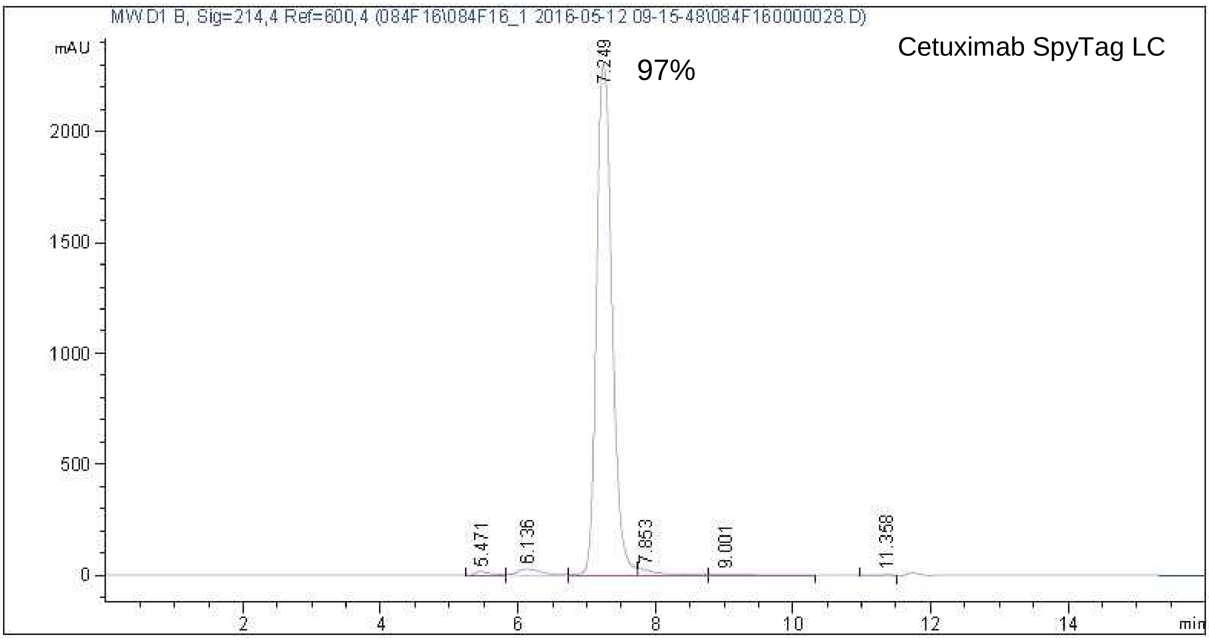
MWD1 B, Sig=214,4 Ref=600,4 (084F16\084F16_1 2016-05-12 09-15-48\084F160000028.D)
mAU
Cetuximab SpyTag LC
97%
7.249
5.471
6.136
7.853
9.001
11.358
2000
1500
1000
500
0
2
4
6
8
10
12
14
min

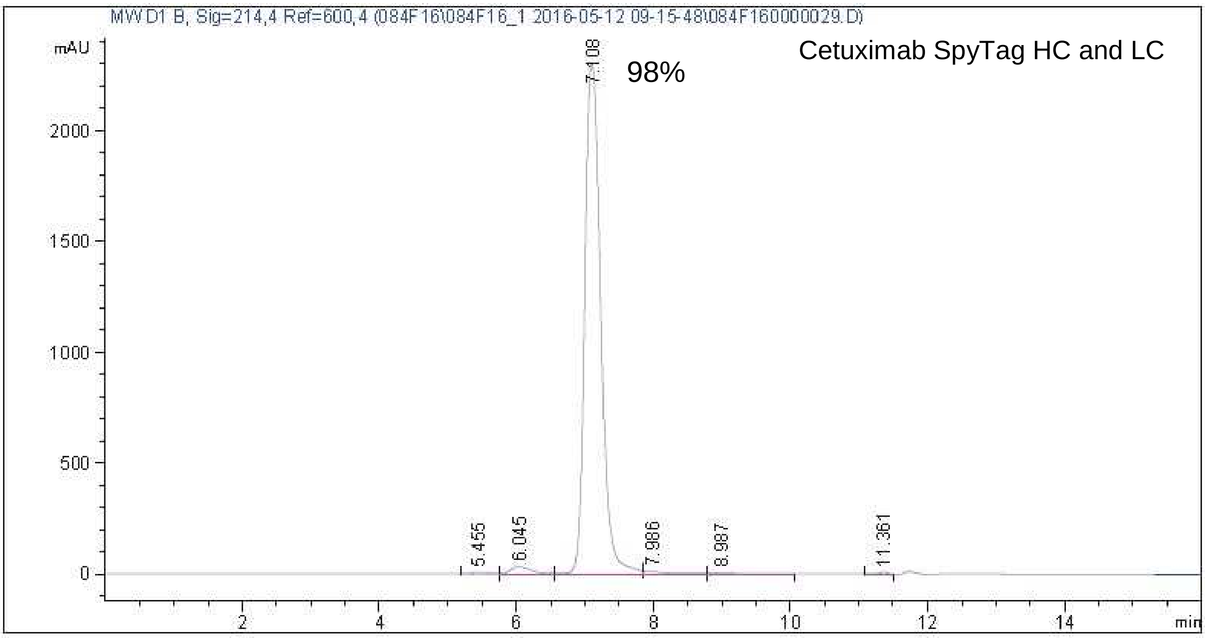
MWD1 B, Sig=214,4 Ref=600,4 (084F16\084F16_1 2016-05-12 09-15-48\084F160000029.D)
mAU
Cetuximab SpyTag HC and LC
98%
7.108
5.455
6.045
7.986
8.987
11.361
2000
1500
1000
500
0
2
4
6
8
10
12
14
min

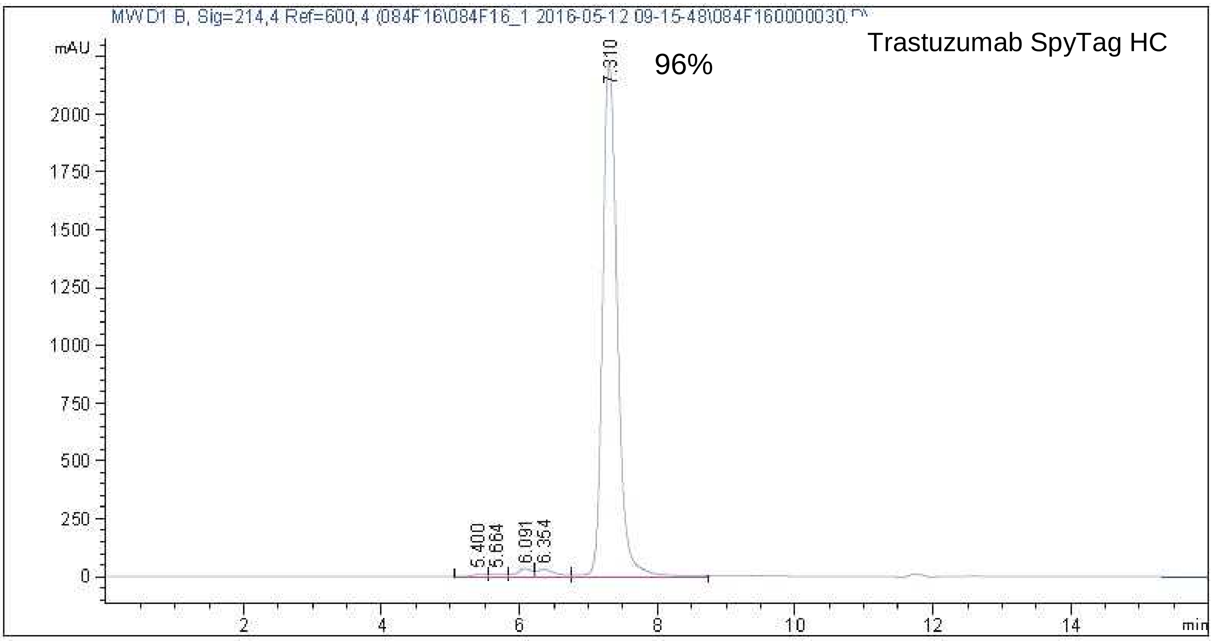

Figure S2. Size exclusion chromatography (SEC) of Spy-tagged cetuximab and trastuzumab variants. Antibodies were analyzed on a TSK Super SW3000 column (Tosoh Bioscience). Cetuximab wildtype showed a monomeric peak with 99% purity whereas Spy-tagged variants showed purities of 96-99% with only minor amounts of aggregates (2-4%).

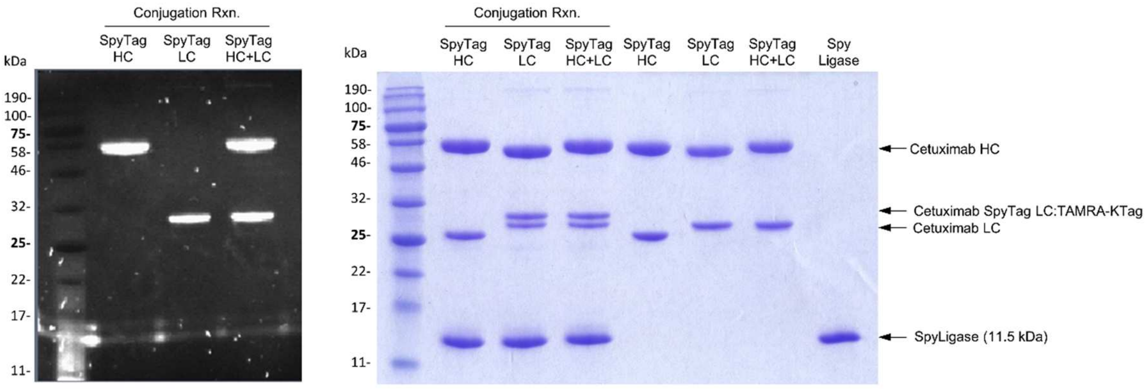

Figure S3. SpyLigase-mediated conjugation reactions of Spy-tagged cetuximab variants with TAMRA-KTag. SDS-PAGE, in-gel fluorescence (left panel), and Coomassie staining (right panel) of the reduced cetuximab-fluorophore conjugates. Reactions were conducted with 3 mol. eq. of SpyLigase and 20-fold excess of TAMRA-KTag.

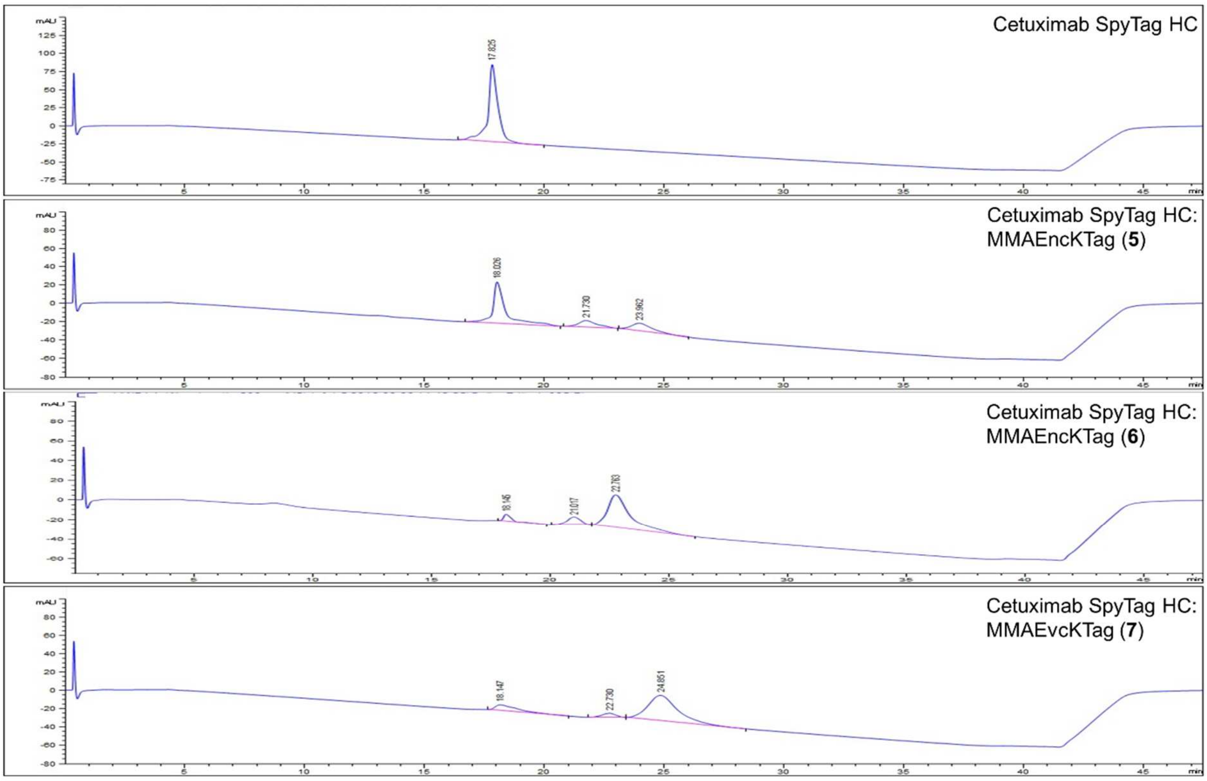

Figure S4. Hydrophobic interaction chromatography (HIC) of SpyLigase mediated antibody-toxin conjugations.

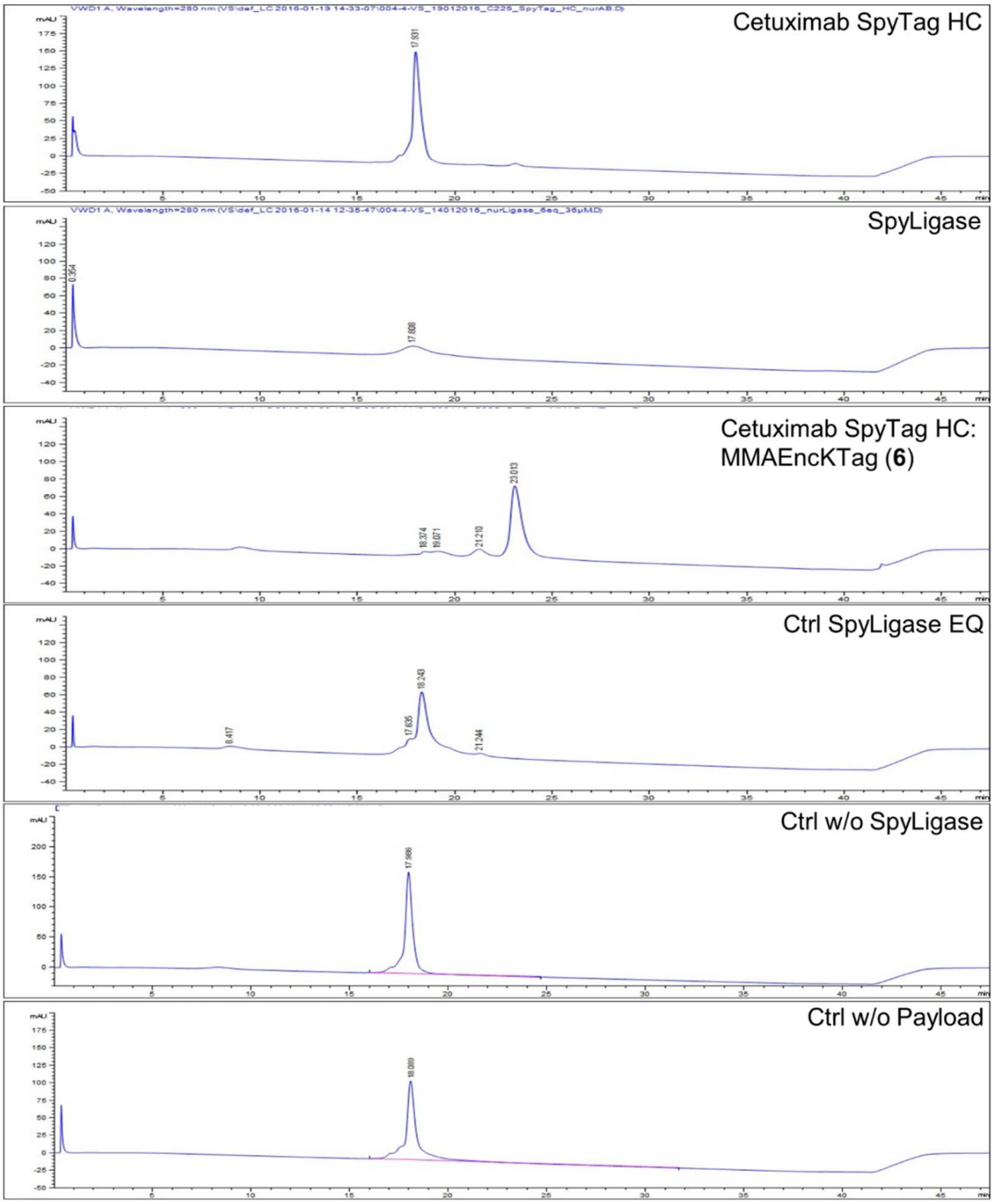

Figure S5. Hydrophobic interaction chromatography (HIC) of control reactions including SpyLigase, Spy-tagged cetuximab, and MMAEncKTag payload **6**.

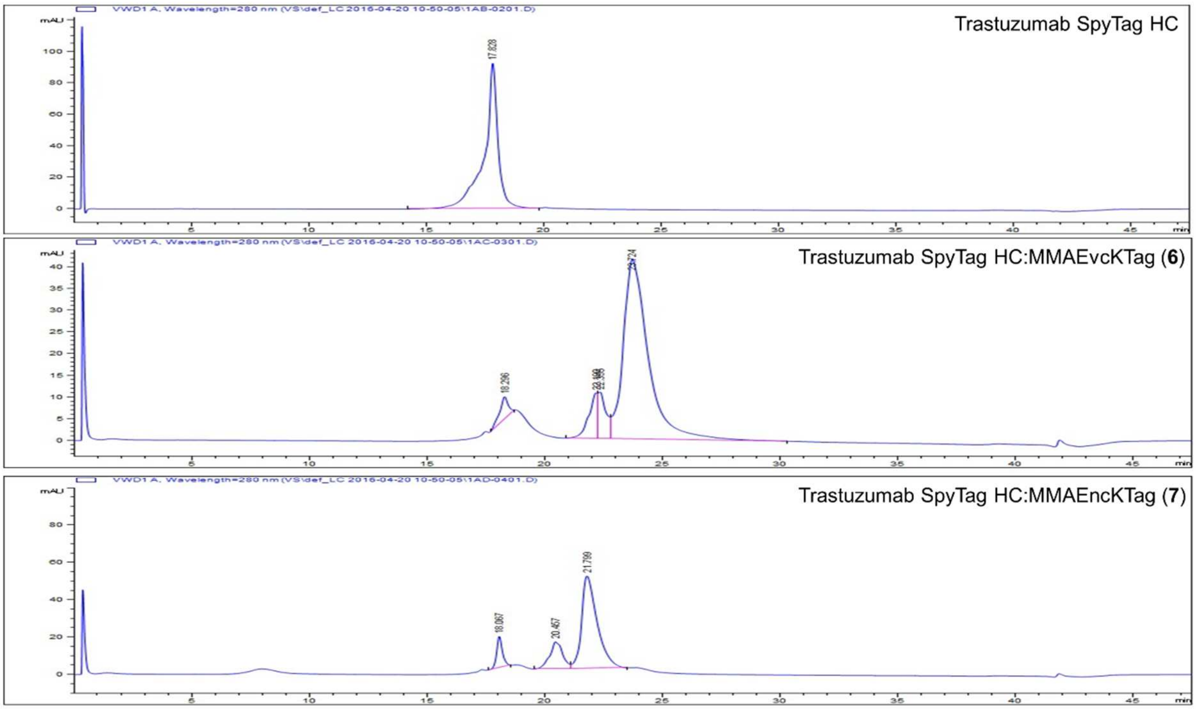

Figure S6. Hydrophobic interaction chromatography (HIC) of SpyLigase mediated conjugation of Spy-tagged trastuzumab with different MMAE-KTag payloads. Conjugation of Spy-tagged trastuzumab with MMAE-payload **6** resulted in a drug-to-antibody ratio (DAR) of about 1.79 whereas conjugation of MMAE-payload **7** to the same antibody resulted in a DAR of about 1.64. Conjugated antibodies were purified by protein A affinity chromatography to remove excess payload and SpyLigase.

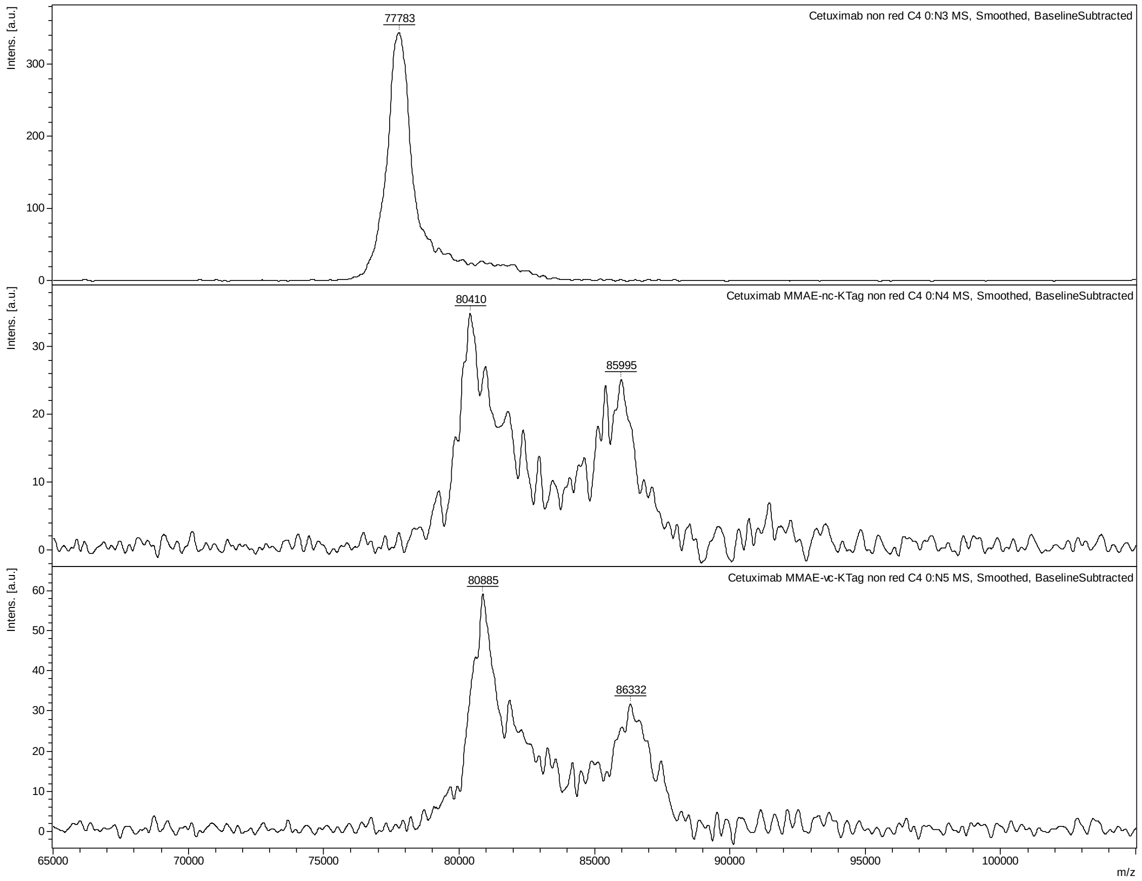

Figure S7. MALDI-MS analysis of intact Spy-tagged cetuximab ADCs. Unmodified Spy-tagged cetuximab exhibited a mass of 77783 Da $[M+2H^+]^{2+}$ (upper panel). Spy-tagged cetuximab conjugated to MMAE-nc-KTag payload **6** showed a mass shift of 2627 Da to 80410 Da, indicating the conjugation to two payloads (calc $[M+2H^+]^{2+}$: 2675 Da) (middle panel). Spy-tagged cetuximab conjugated to MMAE-vc-KTag payload **7** showed a mass shift of 3102 Da to 80885 Da, indicating the conjugation to two payload (calc $[M+2H^+]^{2+}$: 3081 Da) (lower panel).

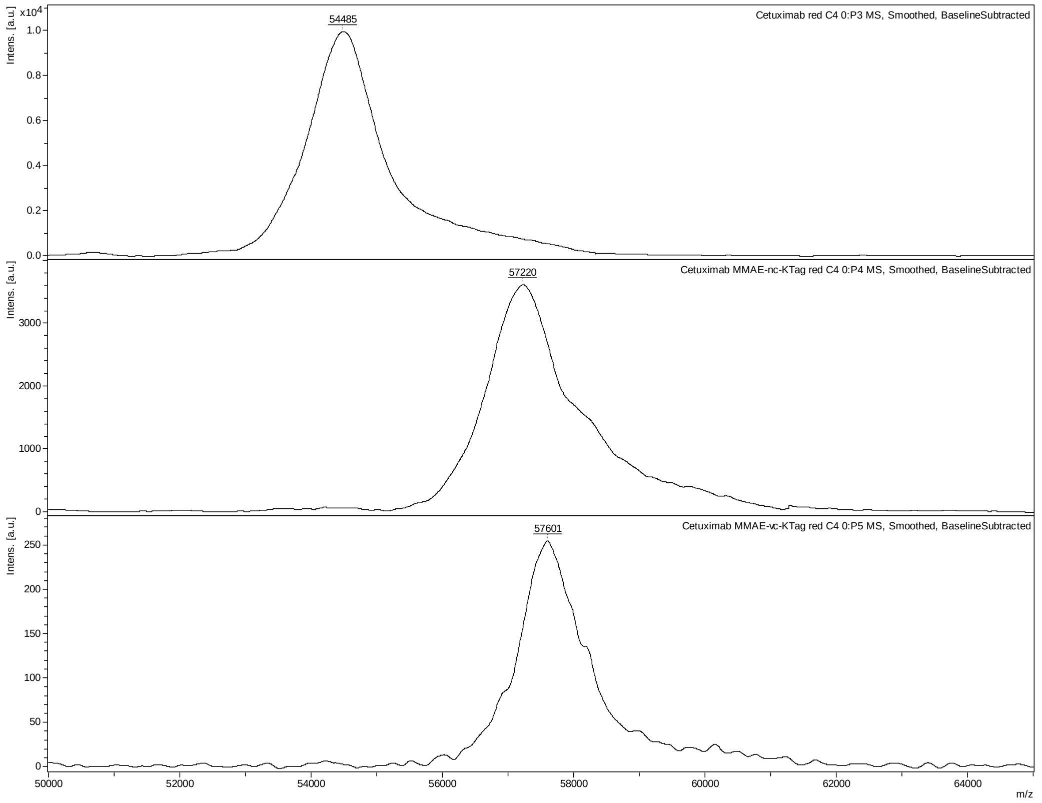

Figure S8. MALDI-MS analysis of reduced Spy-tagged cetuximab ADCs. Unmodified Spy-tagged cetuximab HC indicated a mass of 54485 Da $[M+H^{+}]^{+}$ (upper panel). Spy-tagged cetuximab HC conjugated to MMAE-nc-KTag payload **6** showed a mass shift of 2735 Da to 57220 Da, indicating the conjugation to one payload (calc: 2675 Da) (middle panel). Spy-tagged cetuximab HC conjugated to MMAE-vc-KTag payload **7** showed a mass shift of 3116 Da to 57601 Da, indicating the conjugation to one payload (calc: 3080 Da) (lower panel).

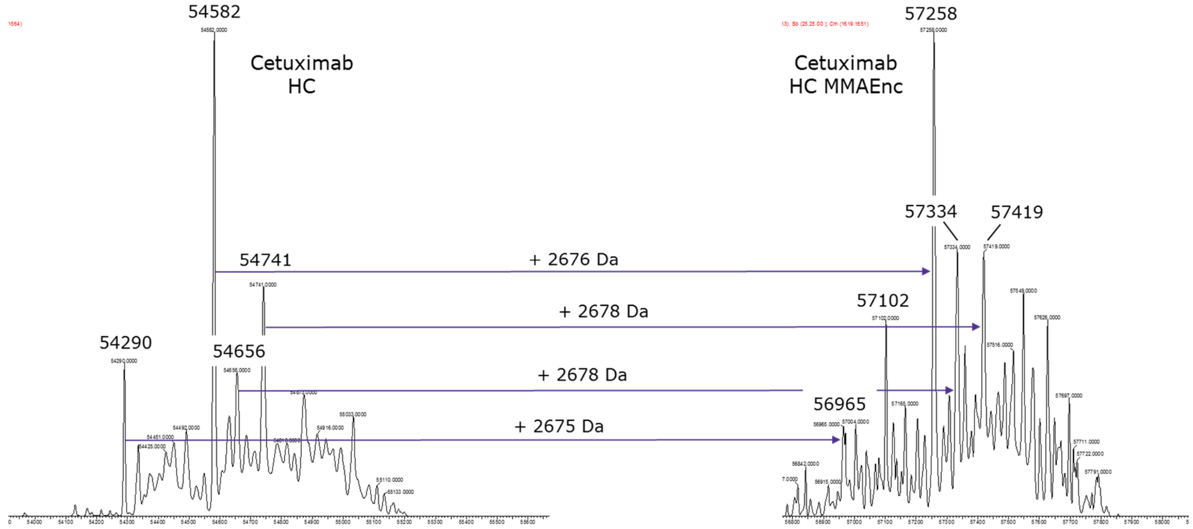

Figure S9. LC-ESI-MS analysis of reduced Spy-tagged cetuximab ADC. Spy-tagged cetuximab HC conjugated to MMAE-nc-KTag payload **6** showed a mass shift of 2676.8 Da (average) indicating the conjugation to one payload (calc: 2675 Da).

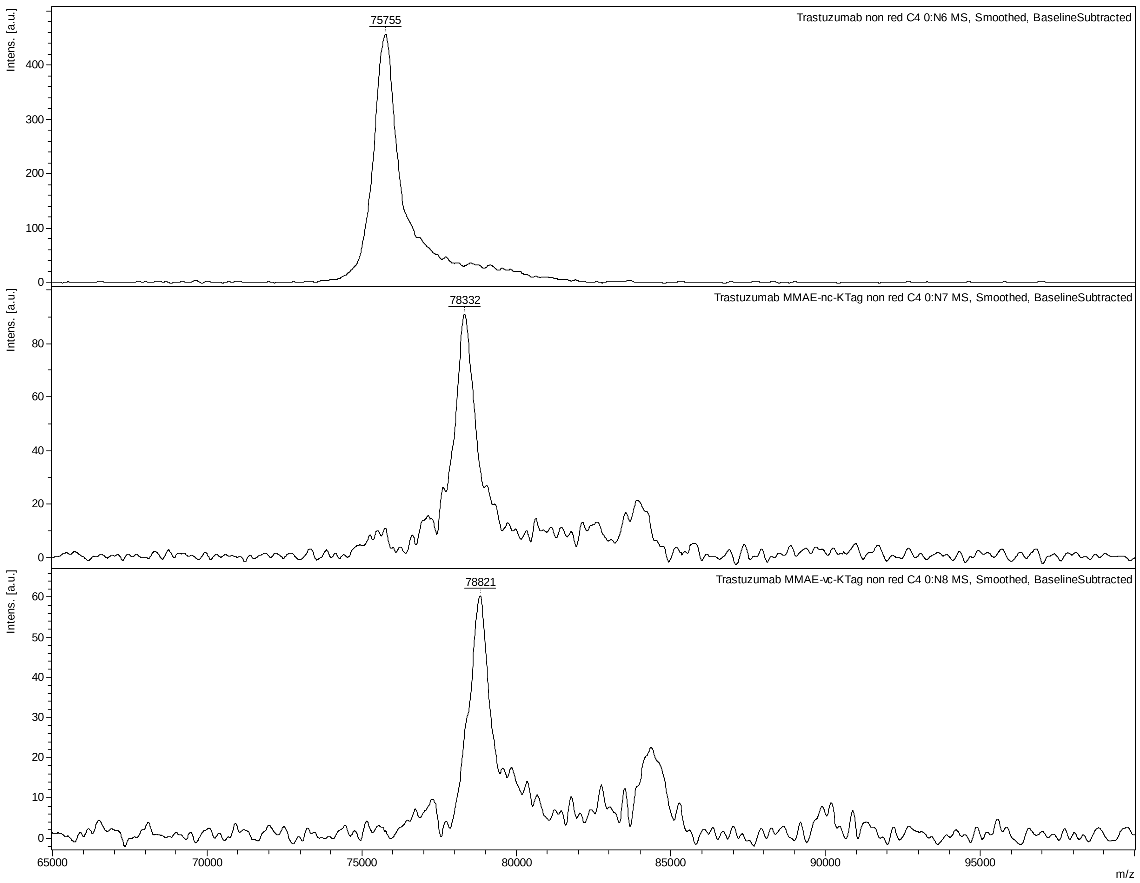

Figure S10. MALDI-MS analysis of intact Spy-tagged trastuzumab ADCs. Unmodified Spy-tagged trastuzumab exhibited a mass of 75755 Da $[M+2H^+]^{2+}$ (upper panel). Spy-tagged trastuzumab conjugated to MMAE-nc-KTag payload **6** showed a mass shift of 2577 Da to 78332 Da, indicating the conjugation to two payloads (calc $[M+2H^+]^{2+}$: 2675 Da) (middle panel). Spy-tagged trastuzumab conjugated to MMAE-vc-KTag payload **7** showed a mass shift of 3066 Da to 78821 Da, indicating the conjugation to two payload (calc $[M+2H^+]^{2+}$: 3080 Da) (lower panel).

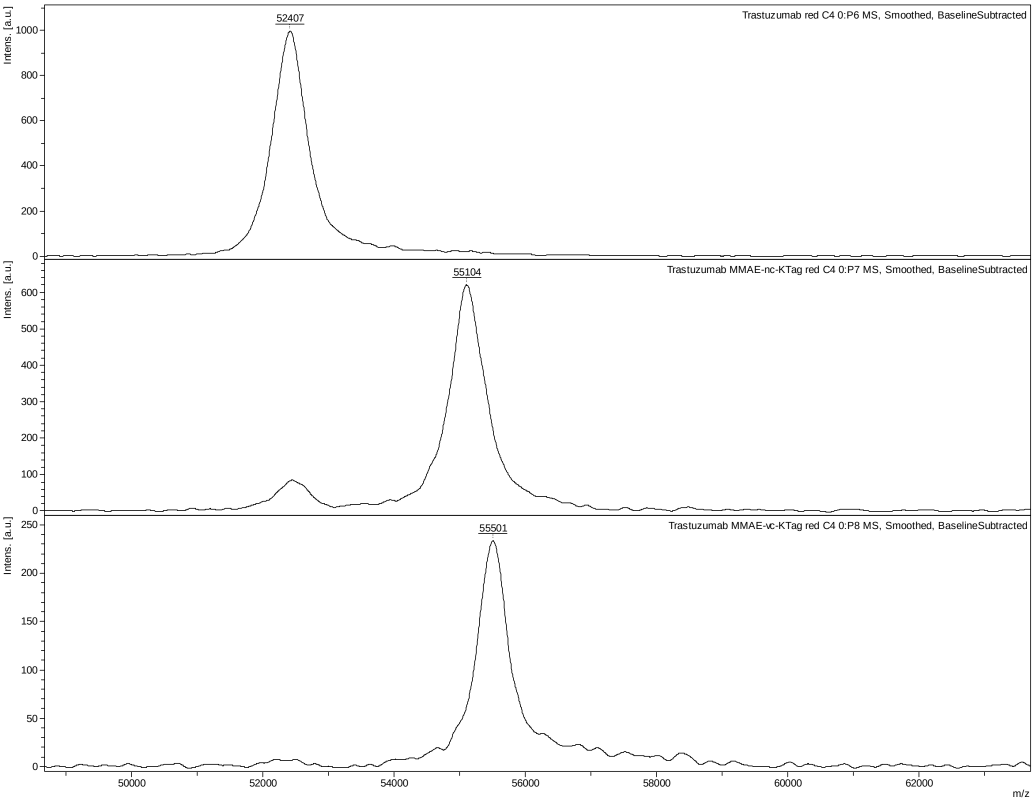

Figure S11. MALDI-MS analysis of reduced Spy-tagged trastuzumab ADCs. Unmodified Spy-tagged cetuximab HC exhibited a mass of 52407 Da $[M+H^+]^+$ (upper panel). Spy-tagged trastuzumab HC conjugated to MMAE-nc-KTag payload **6** showed a mass shift of 2697 Da to 55104 Da, indicating the conjugation to one payload (calc: 2675 Da) (middle panel). Spy-tagged trastuzumab HC conjugated to MMAE-vc-KTag payload **7** showed a mass shift of 3094 Da to 55501 Da, indicating the conjugation to one payload (calc: 3080 Da) (lower panel).

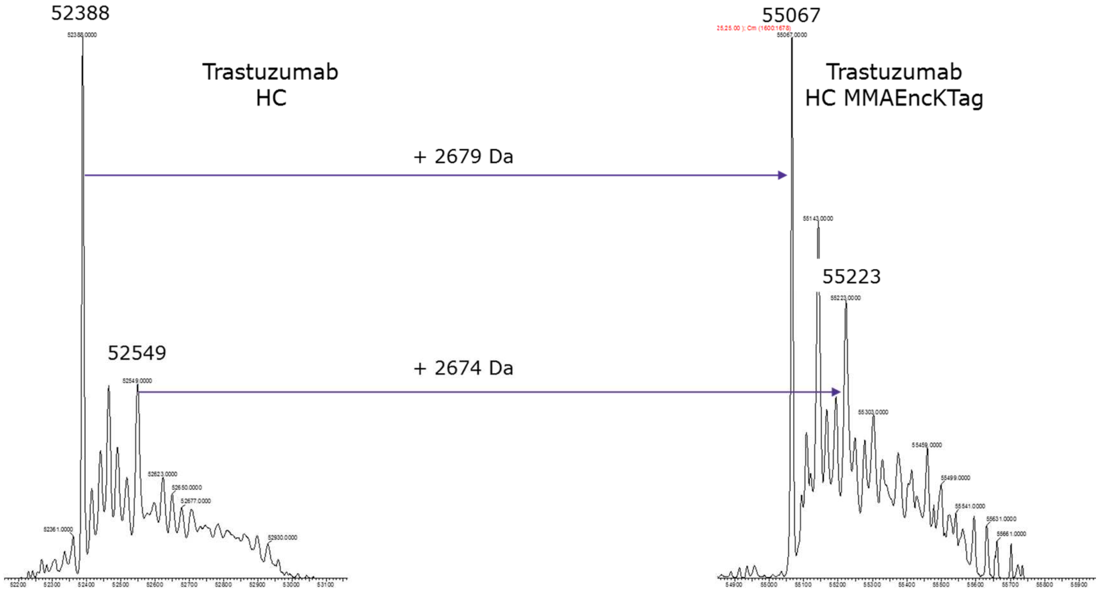

Figure S12. LC-ESI-MS analysis of reduced Spy-tagged trastuzumab ADC. Spy-tagged trastuzumab HC conjugated to MMAE-nc-KTag payload **6** showed a mass shift of 2676.5 Da (average) indicating the conjugation to one payload (calc: 2675 Da).

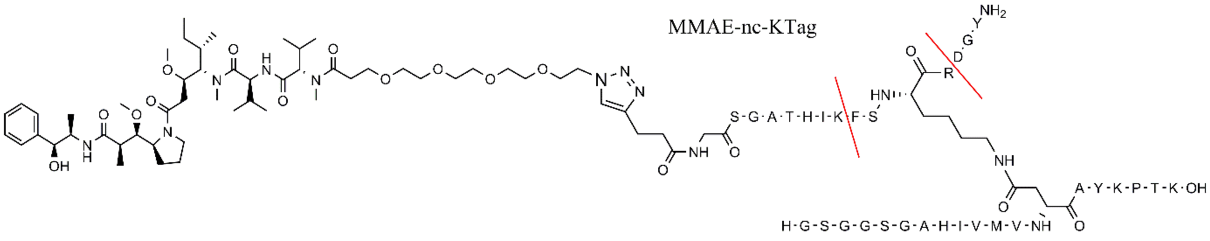

Cetuximab *C*-terminal
SpyTag fragment

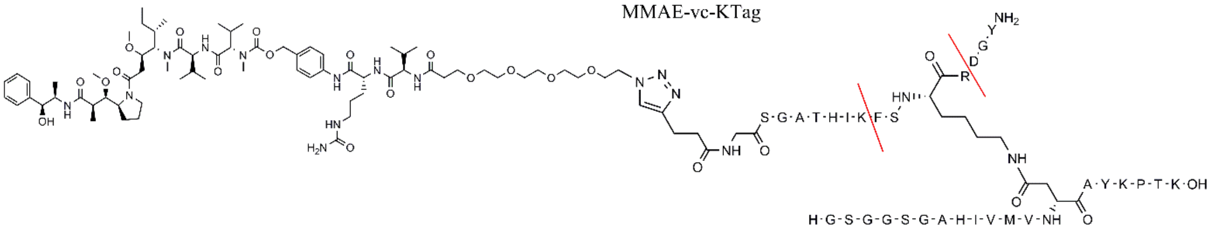

Cetuximab *C*-terminal
SpyTag fragment

(-SGATHIK)FSKR(DGY)

-GSGGSGAHIVMVDAYKPT(K)

$[M+H^+]^+$ (without brackets): 2265.1444

Figure S13. Chemical structures of trypsin-digested conjugation sites of Spy-tagged cetuximab ADCs. Trypsin cleavage sites are indicated as red lines. The resulting fragment of SpyTag covalently linked to KTag via isopeptide bond between lysine and aspartate residues with an expected mass of 2264.1444 $[M+H^+]^+$ is shown.

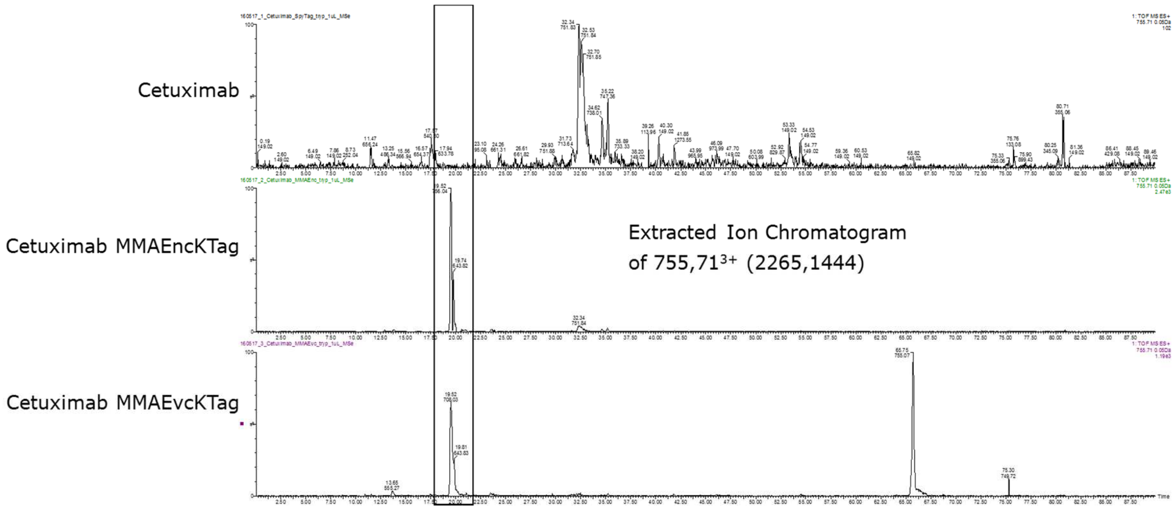

Figure S14. LC-ESI-MS analysis of trypsinated Spy-tagged cetuximab ADCs. Ions corresponding to the conjugated *C*-terminal Spy-Tag fragments with a calculated monoisotopic mass of 2265.1444 (measured 755.71 $[M+3H^{+}]^{+3}$) were extracted (boxed). The fragment was not observed in the unconjugated cetuximab control (upper panel).

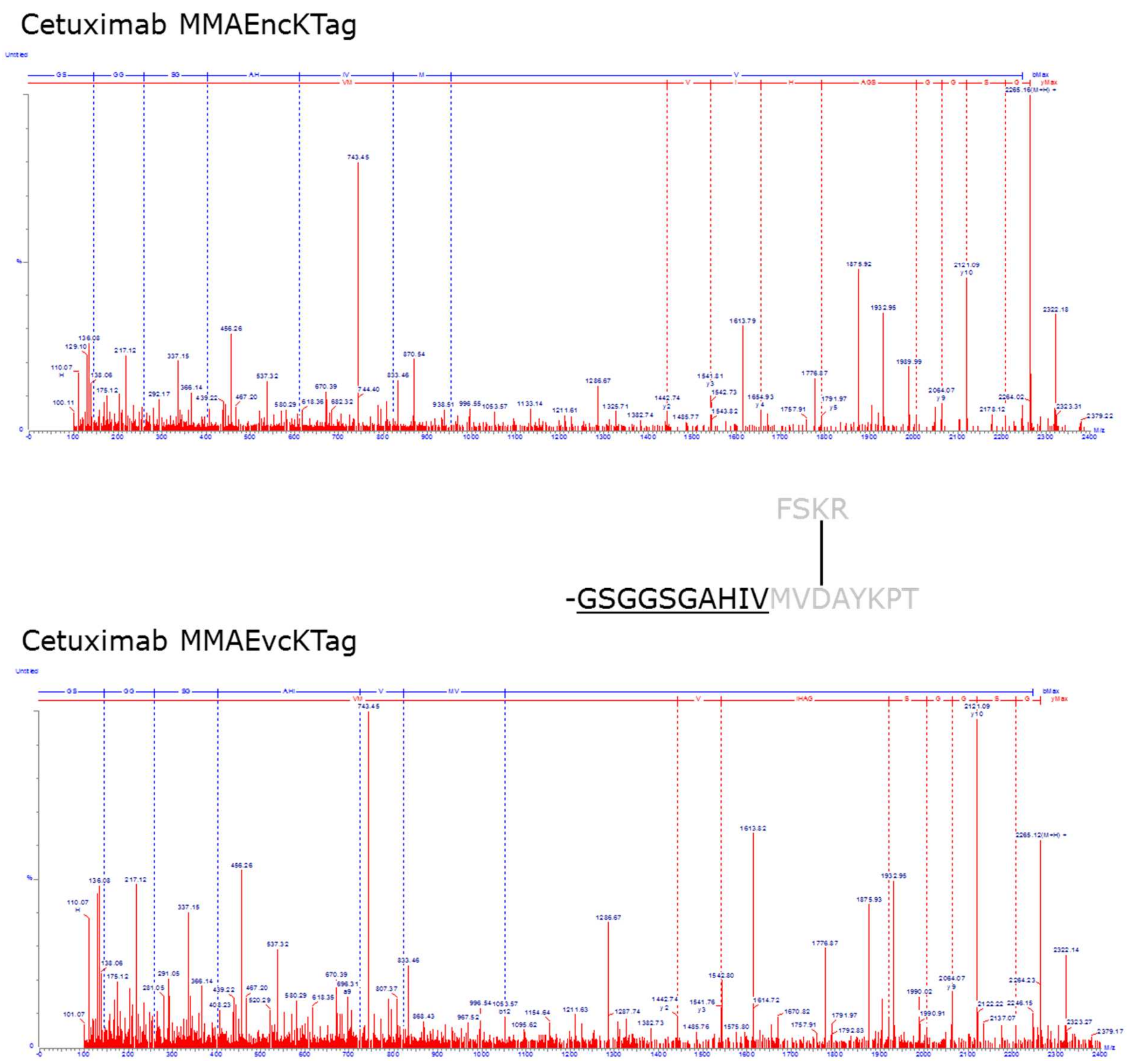

Figure S15. Peptide-map of trypsinated Spy-tagged cetuximab ADCs. The fragment corresponding to SpyTag covalently linked to KTag via isopeptide bond between lysine and aspartate residues was partially sequence verified by LC-ESI-MSe. The identified *N*-terminal part of the fragment is indicated underlined in black.

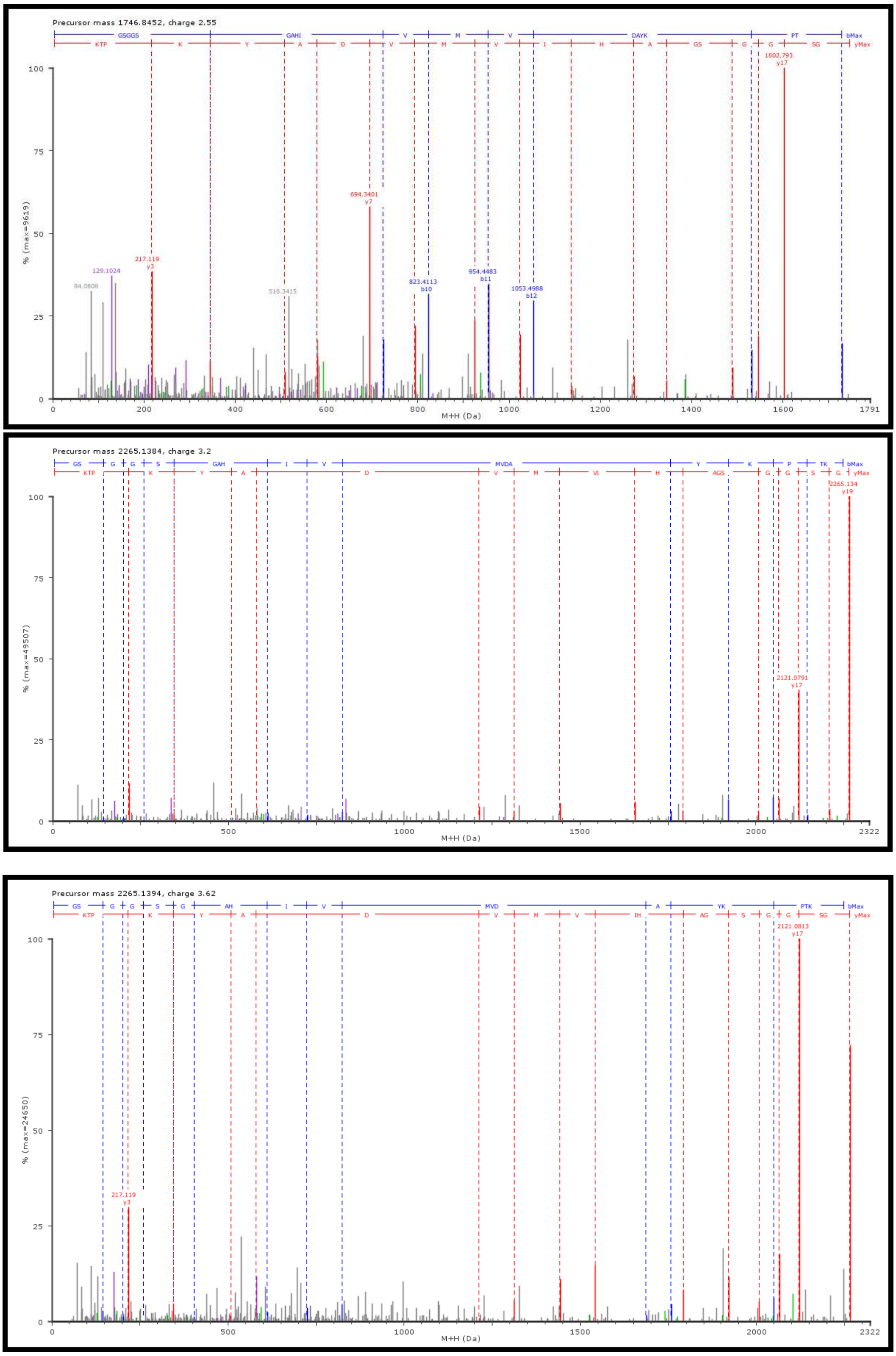

Figure S16. Peptide-map of trypsinated Spy-tagged cetuximab ADCs. The fragment corresponding to SpyTag covalently linked to KTag via isopeptide bond between lysine and aspartate residues was partially sequence verified by LC-ESI-MSe. The gap at the aspartate residues of the obtained y-ion deriving from SpyTag indicated the attachment of the variable modification FSKR resulting from the KTag-payloads **6** (middle panel) and **7** (lower panel). This gap was not observed in the unmodified Spy-tagged cetuximab control (upper panel).

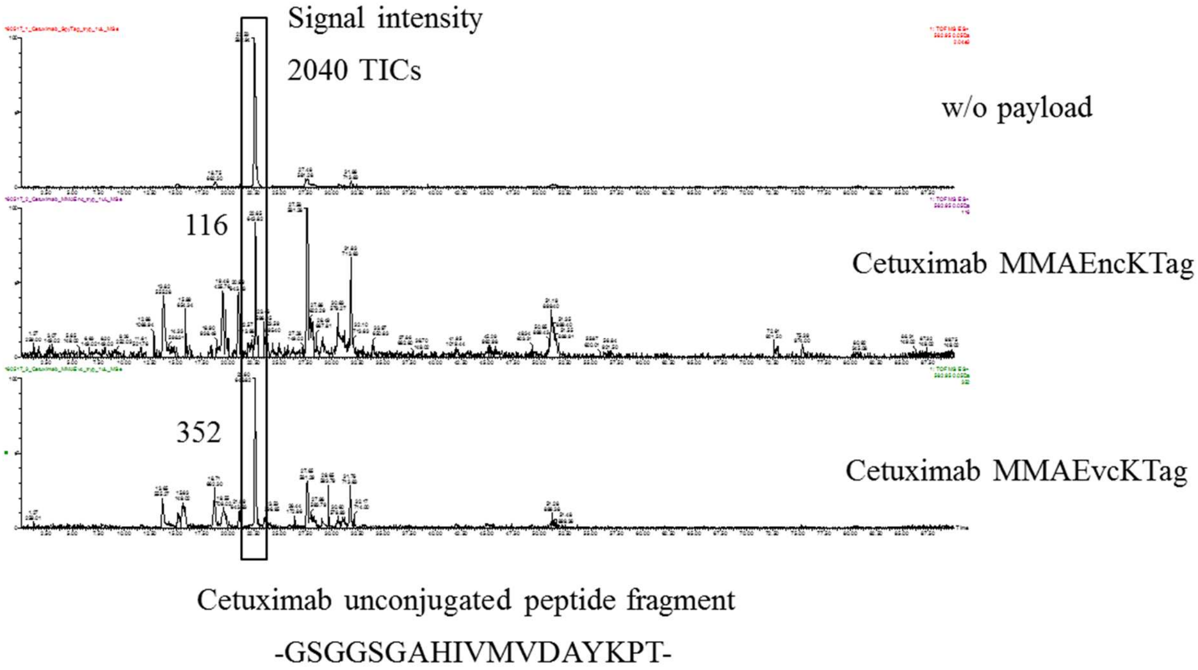

Figure S17. LC-ESI-MS quantification of unconjugated Spy-Tag peptide fragment. Spy-tagged cetuximab (upper panel, w/o payload) and Spy-tagged cetuximab ADCs (middle and lower panel) were digested with trypsin and the signal intensities of unconjugated Spy-Tag fragment analyzed (black box).

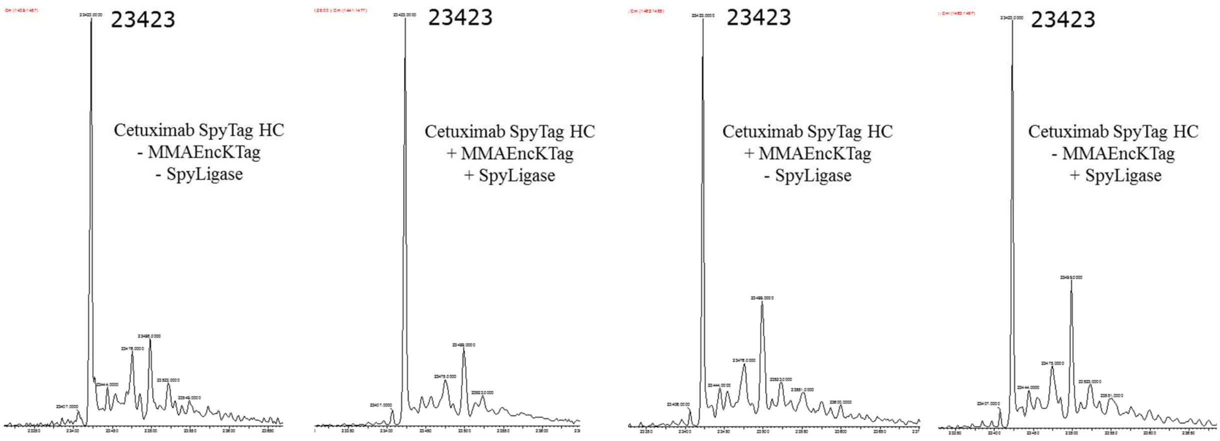

Figure S18. LC-ESI-MS analysis of reduced cetuximab LCs. The light chain of Spy-tagged cetuximab (Cetuximab SpyTag HC -MMAEncKTag -SpyLigase), Spy-tagged cetuximab ADC (Cetuximab SpyTag HC +MMAEncKTag +SpyLigase), and control reactions without SpyLigase (Cetuximab SpyTag HC +MMAEncKTag –SpyLigase) or without payload (Cetuximab SpyTag HC -MMAEncKTag +SpyLigase) were analyzed. No unspecific conjugation of the antibody light chain was observed.

Analytics of peptides and payloads

GSG-KTag-GY (GSGATHIKFSKRDGY)

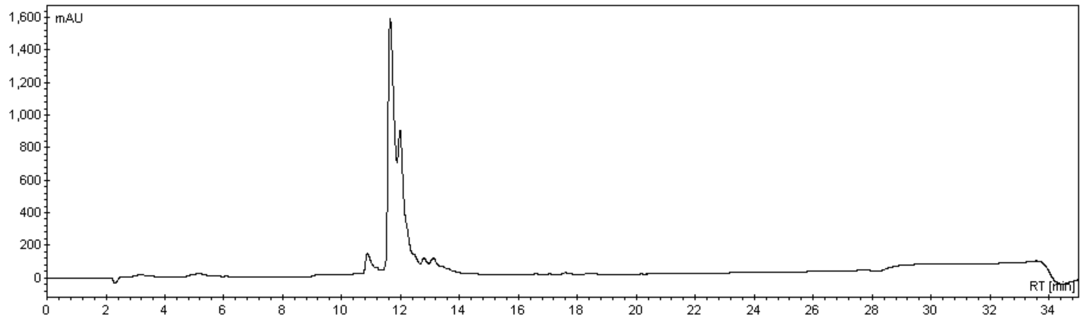

Figure S19. RP-HPLC analysis of GSG-KTag-GY (gradient 10 to 80).

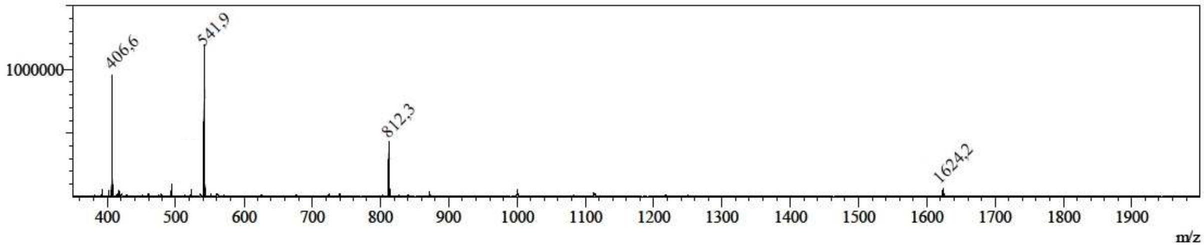

Figure S20. LC-MS analysis of GSG-KTag-GY, calc. mass: 1622.78 g/mol; meas. m/z: 1624.2 $[M+H]^{+}$, 812.3 $[M+2H]^{2+}$, 541.9 $[M+3H]^{3+}$, 406.6 $[M+4H]^{4+}$.

Compound **1**: TAMRA-GSG-KTag-GY

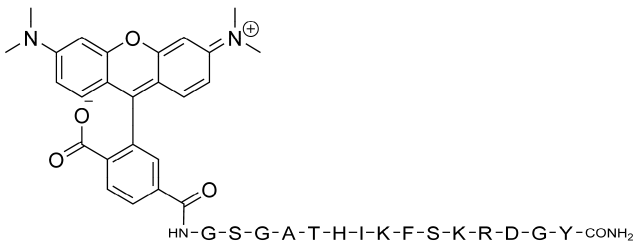

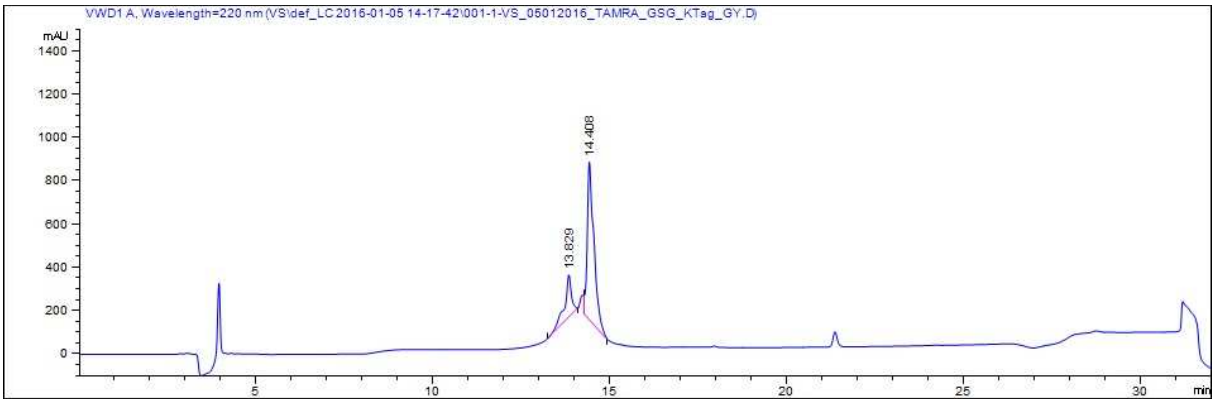

Figure S21. RP-HPLC analysis of compound **1**, mixture of isomers (gradient 10 to 80).

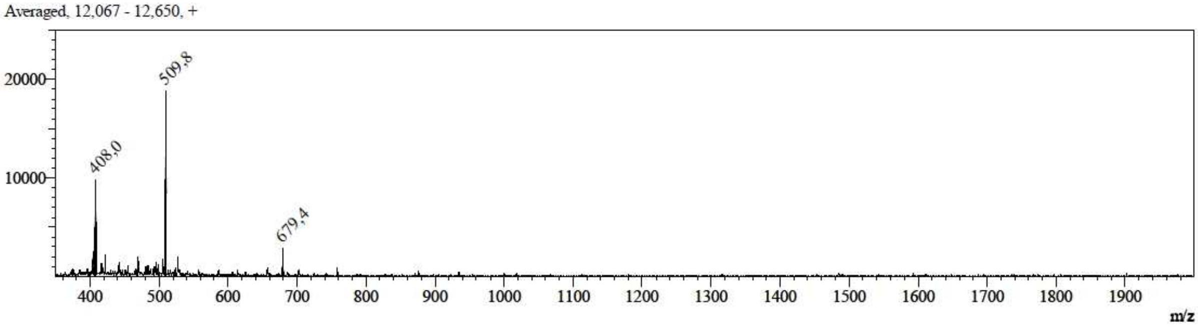

Figure S22. LC-MS analysis of compound **1**, calc. mass: 2035.22 g/mol; meas. m/z: 679.4 $[M+3H]^{3+}$, 509.8 $[M+4H]^{4+}$, 408.0 $[M+5H]^{5+}$.

GSG-KTag-GY with *N*-terminal propargylacetic acid moiety

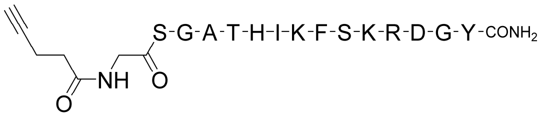

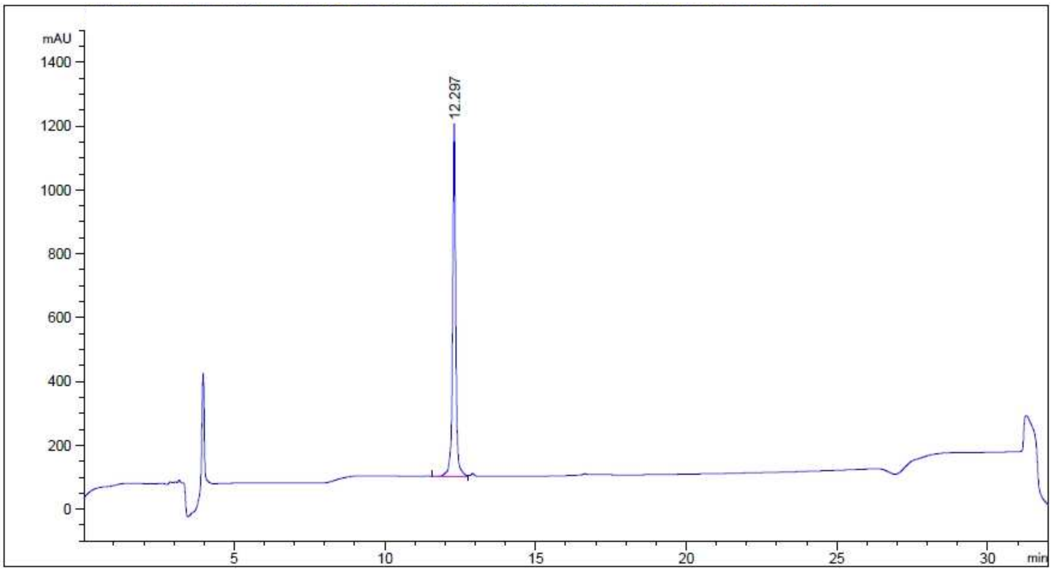

Figure S23. RP-HPLC analysis of GSG-KTag-GY with *N*-terminal propargylacetic acid moiety (gradient 10 to 80).

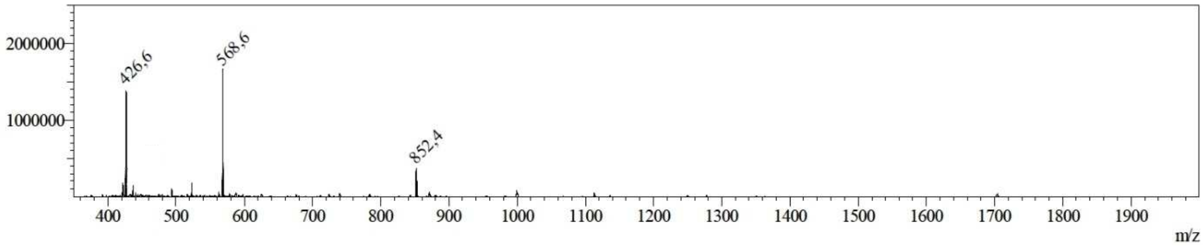

Figure S24. LC-MS analysis of GSG-KTag-GY with *N*-terminal propargylacetic acid moiety, calc. mass: 1702.87 g/mol; meas. m/z: 852.4 $[M+2H]^{2+}$, 568.6 $[M+3H]^{3+}$, 426.6 $[M+4H]^{4+}$.

<u>Compound **2**: Biotin-GSG-KTag-GY</u>

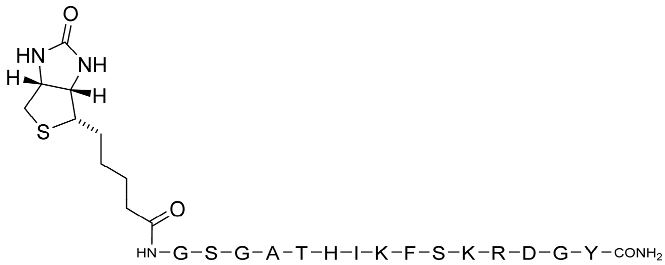

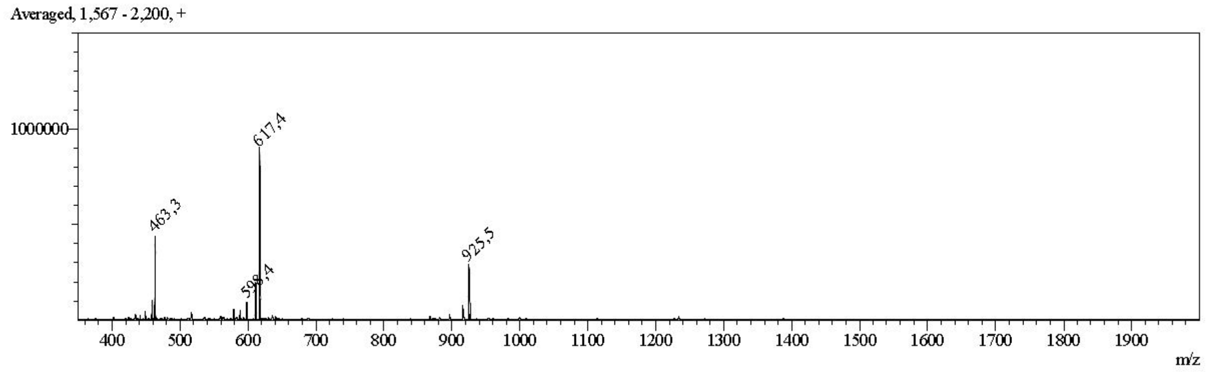

Figure S25. LC-MS analysis of compound **2**, calc. mass: 1849.10 g/mol; meas. m/z: 925.5 $[M+2H]^{2+}$, 617.4 $[M+3H]^{3+}$, 463.3 $[M+4H]^{4+}$.

<u>GSG-SpyTag (GSGAHIVMVDAYKPTK)</u>

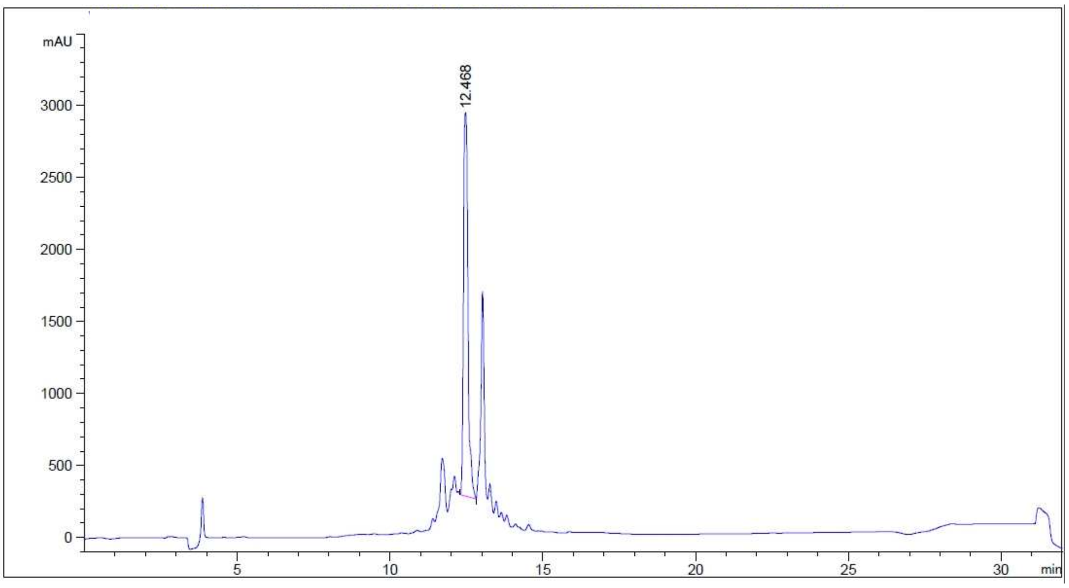

Figure S26. RP-HPLC analysis of GSG-SpyTag (gradient 10 to 80).

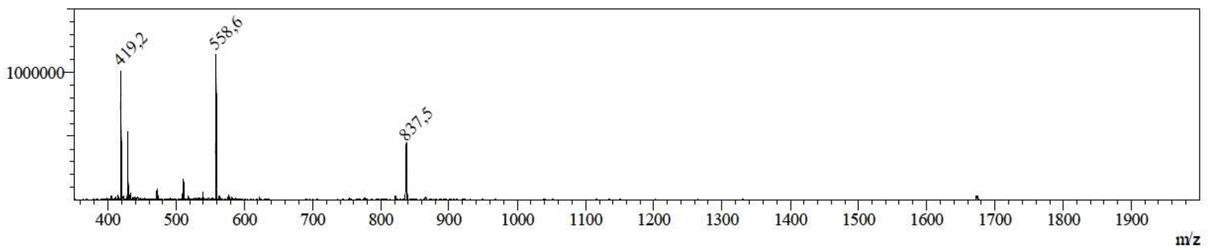

Figure S27. LC-MS analysis of GSG-SpyTag, calc. mass: 1672.95 g/mol; meas. m/z: 837.5 $[M+2H]^{2+}$, 558.6 $[M+3H]^{3+}$, 419.2 $[M+4H]^{4+}$.

Compound **3**: TAMRA-GSG-SpyTag

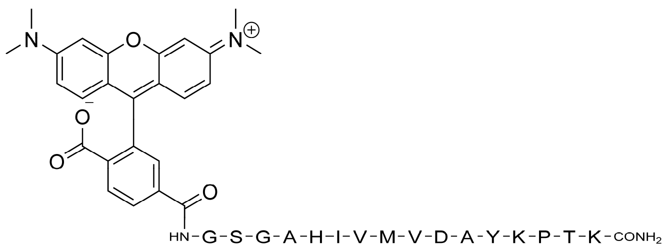

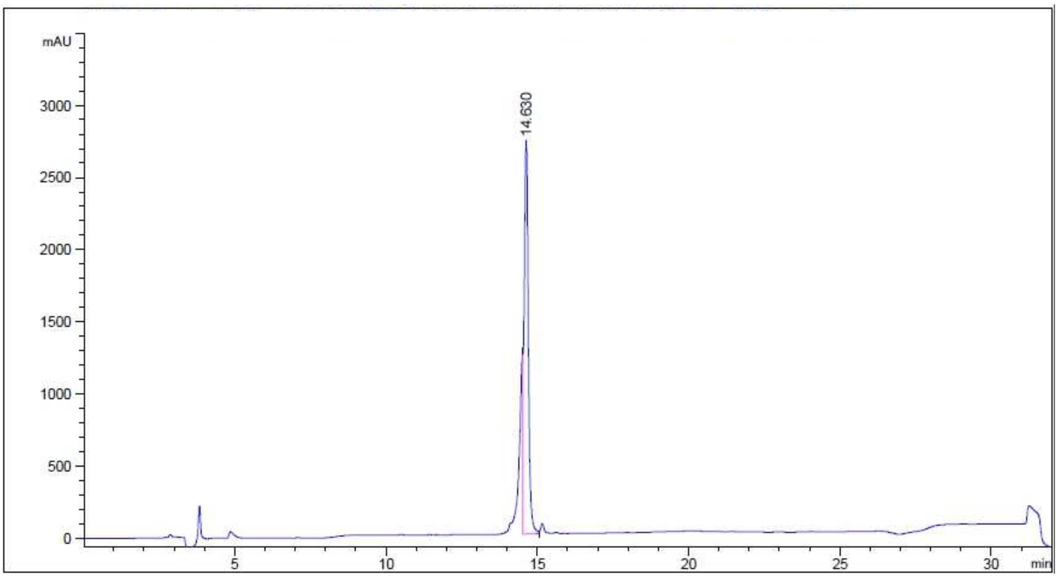

Figure S28. RP-HPLC analysis of compound **3**, isomer 1 (gradient 10 to 80).

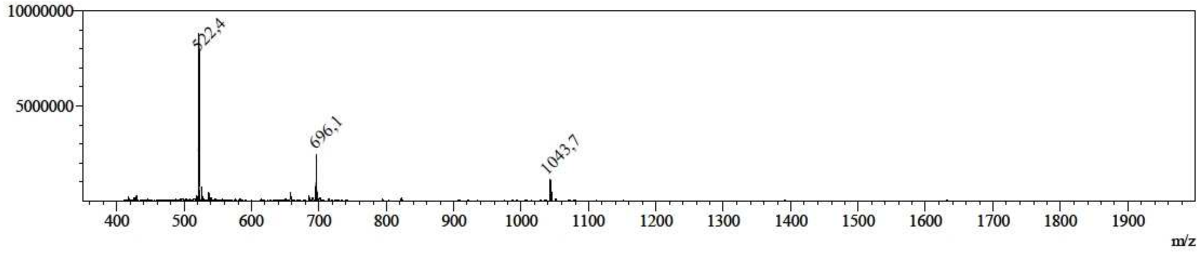

Figure S29. LC-MS analysis of compound **3**, calc. mass: 2085.38 g/mol; meas. m/z: 1043.7 $[M+2H]^{2+}$, 696.1 $[M+3H]^{3+}$, 522.4 $[M+4H]^{4+}$.

Compound **4:** Biotin-SpyTag

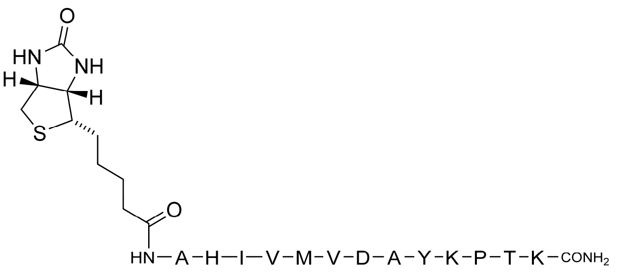

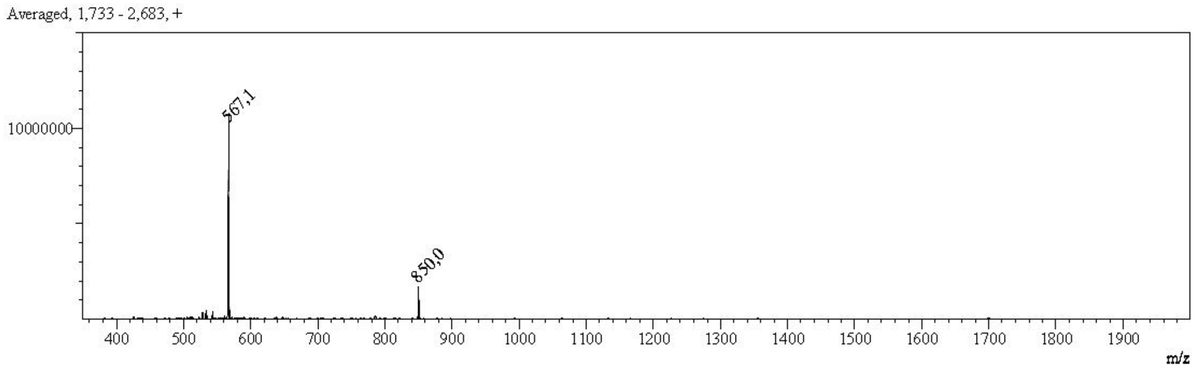

Figure S30. LC-MS analysis of compound **4**, calc. mass: 1697.07 g/mol; meas. m/z: 850.0 $[M+2H]^{2+}$, 567.1 $[M+3H]^{3+}$.

Toxin 1: Monomethyl auristatin E (MMAE) with non-cleavable linker (nc) and carboxy functionality

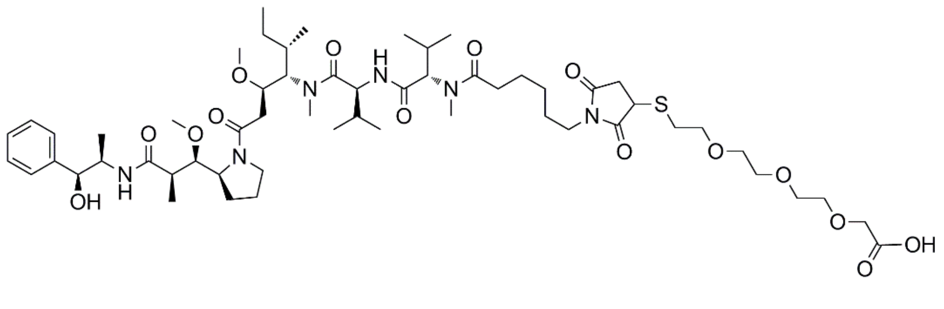

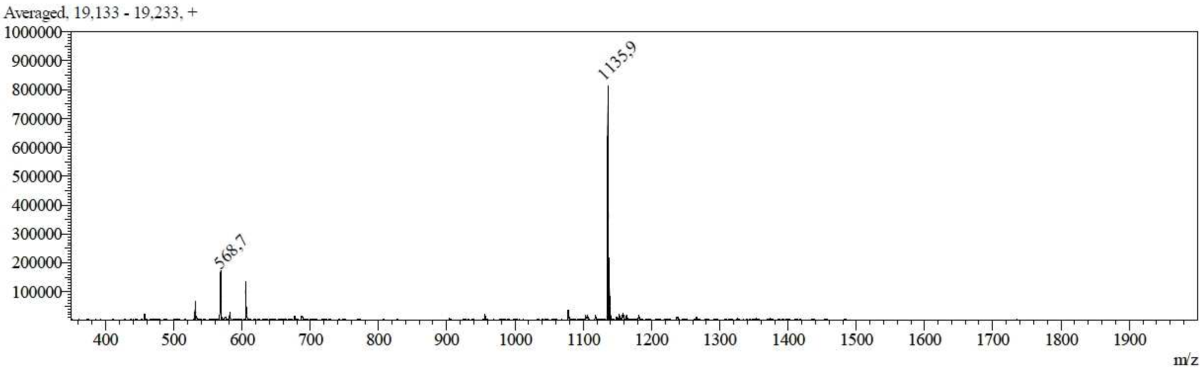

Figure S31. LC-MS analysis of toxin 1, calc. mass: 1135.45 g/mol; meas. m/z: 1135.9 $[M+1H]^{1+}$, 568.7 $[M+2H]^{2+}$.

Compound **5**: MMAE-nc-KTag (Amide bond)

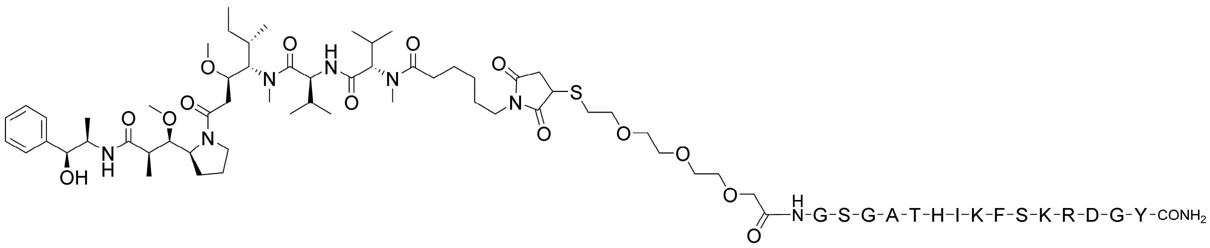

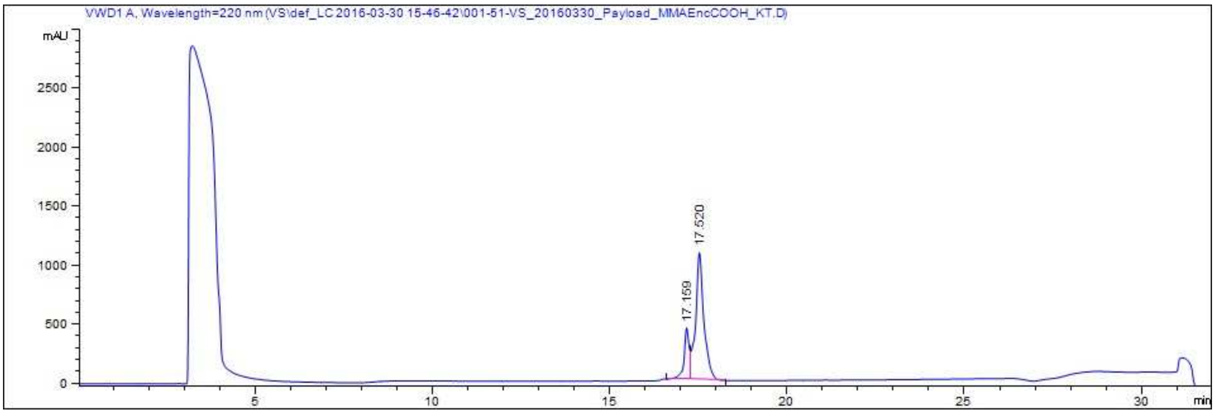

Figure S32. RP-HPLC analysis of compound **5** (gradient 10 to 80).

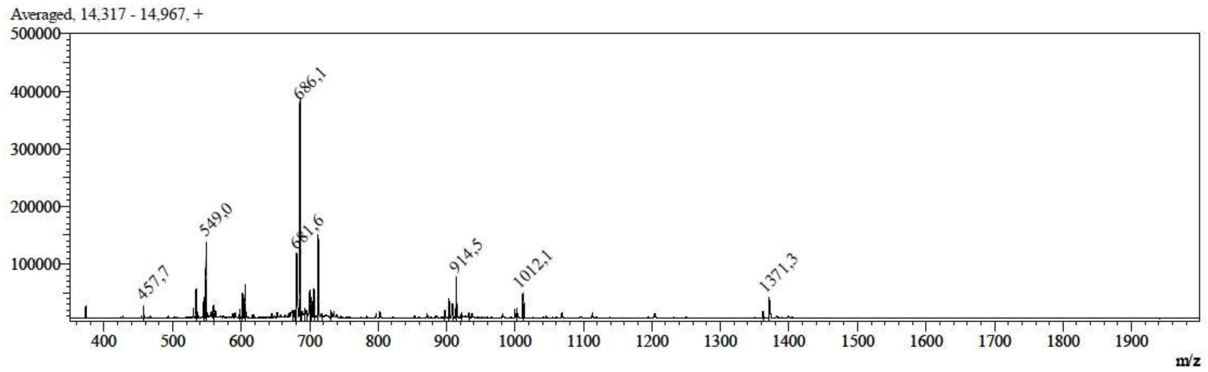

Figure S33. LC-MS analysis of compound **5**, calc. mass: 2740.22 g/mol; meas. m/z: 1371.3 $[M+2H]^{2+}$, 914.5$[M+3H]^{3+}$, 686.1 $[M+4H]^{4+}$,549.0 $[M+5H]^{5+}$.

Toxin 2: Monomethyl auristatin E (MMAE) with non-cleavable linker (nc) and azide functionality

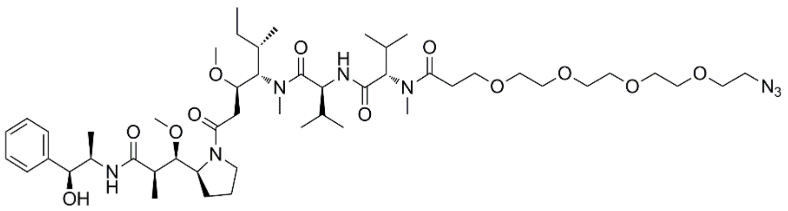

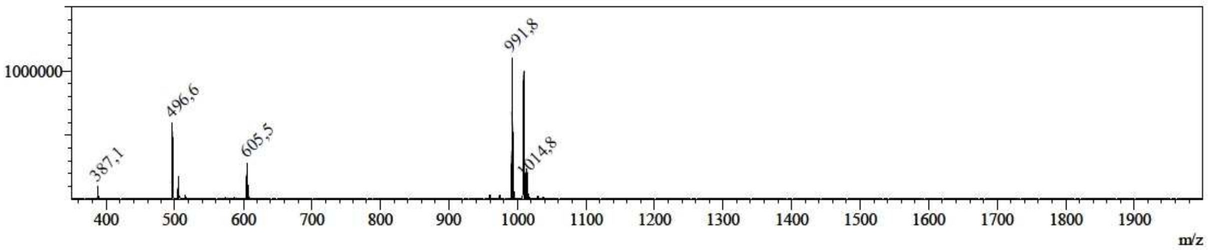

Figure S34. LC-MS analysis of toxin 2, calc. mass: 991.26 g/mol; meas. m/z: 991.8 $[M+1H]^{1+}$, 469.6 $[M+2H]^{2+}$.

Compound **6**: MMAE-nc-KTag (click)

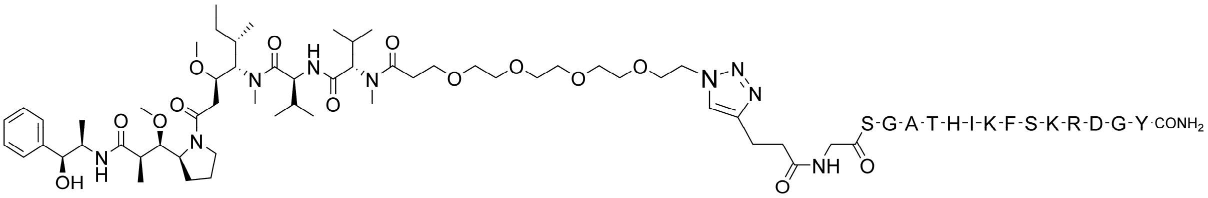

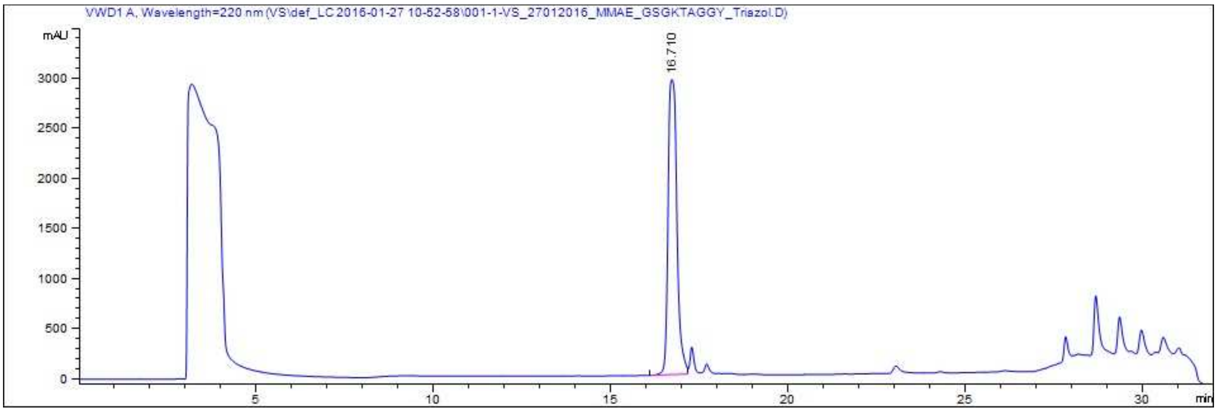

Figure S35. RP-HPLC analysis of compound **6** (gradient 10 to 80).

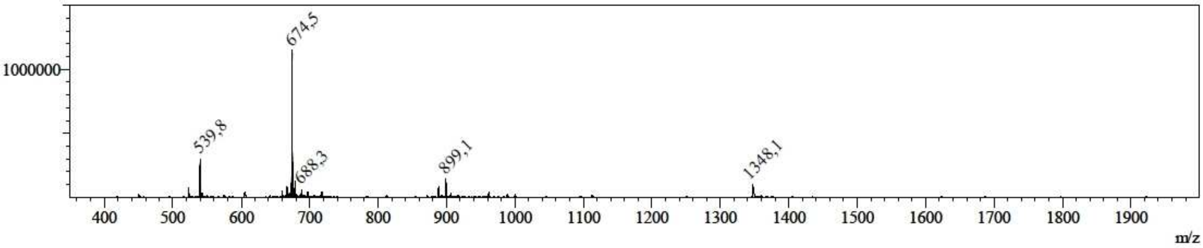

Figure S36. LC-MS analysis of compound **6**, calc. mass: 2694.18 g/mol; meas. m/z: 1348.1$[M+2H]^{2+}$, 899.1 $[M+3H]^{3+}$, 674.5$[M+4H]^{4+}$, 539.8$[M+5H]^{5+}$.

<u>Toxin 3: Monomethyl auristatin E (MMAE) with cleavable vc-PABC linker (vc) and azide functionality</u>

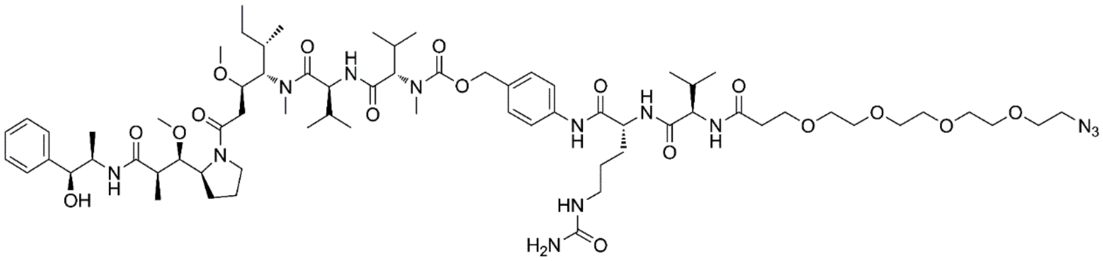

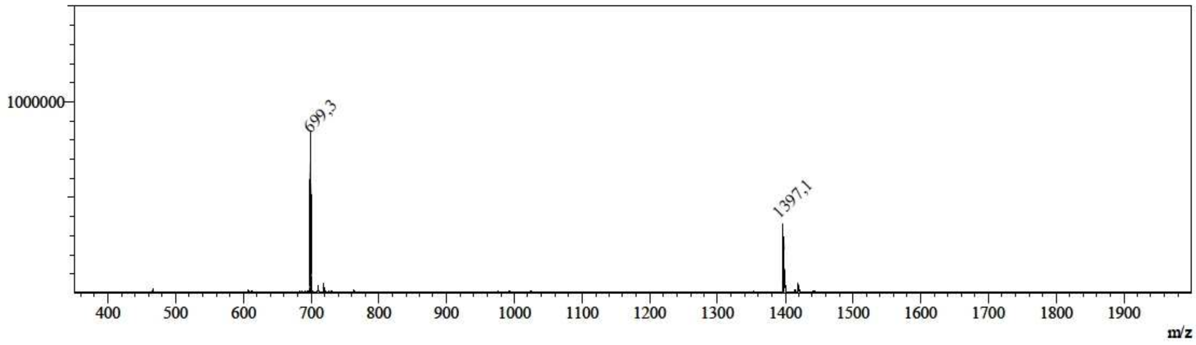

Figure S37. LC-MS analysis of toxin 3, calc. mass: 1396.71 g/mol; meas. m/z: 1397.7 $[M+1H]^{1+}$, 699.3 $[M+2H]^{2+}$.

<u>Compound **7**: MMAE-vc-KTag (click)</u>

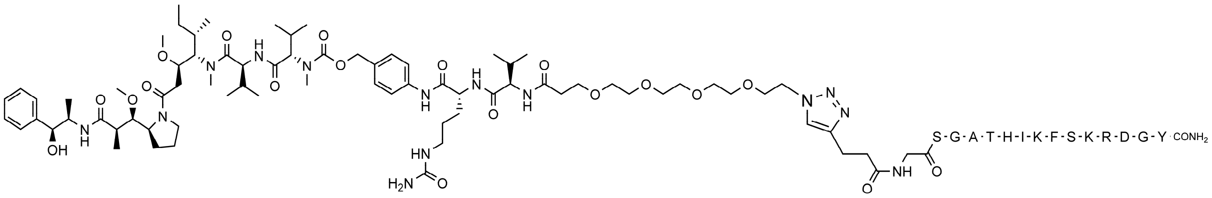

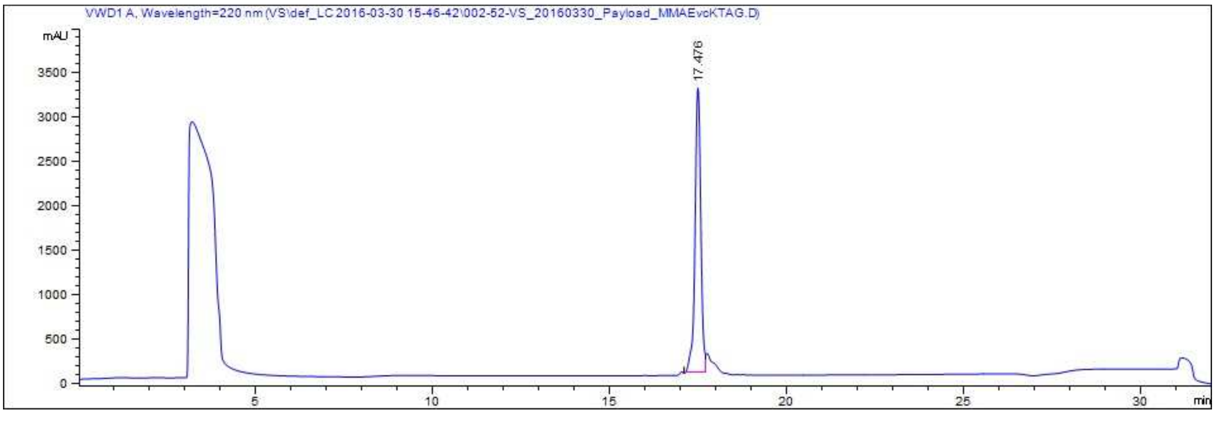

Figure S38. RP-HPLC analysis of compound **7** (gradient 10 to 80).

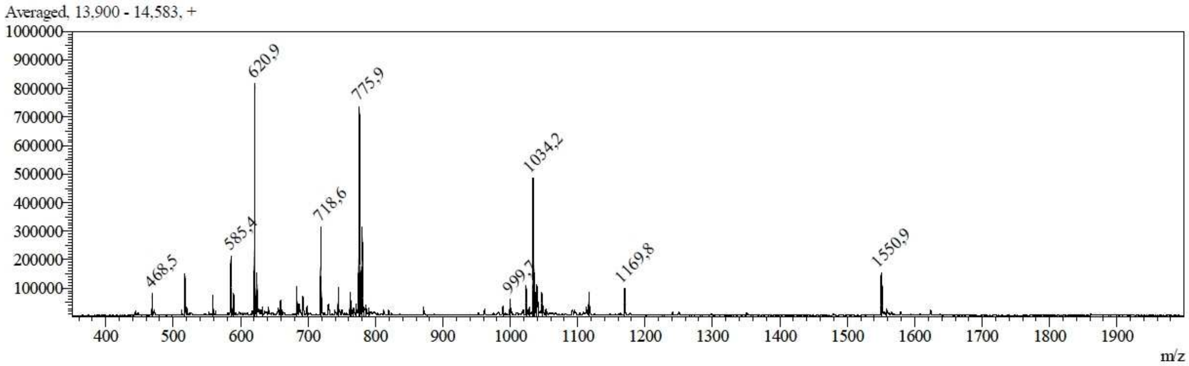

Figure S39. LC-MS analysis of compound **7**, calc. mass: 3099.63 g/mol; meas. m/z: 1550.9 $[M+2H]^{2+}$, 1034.2 $[M+3H]^{3+}$, 775.9 $[M+4H]^{4+}$, 620.9 $[M+5H]^{5+}$.

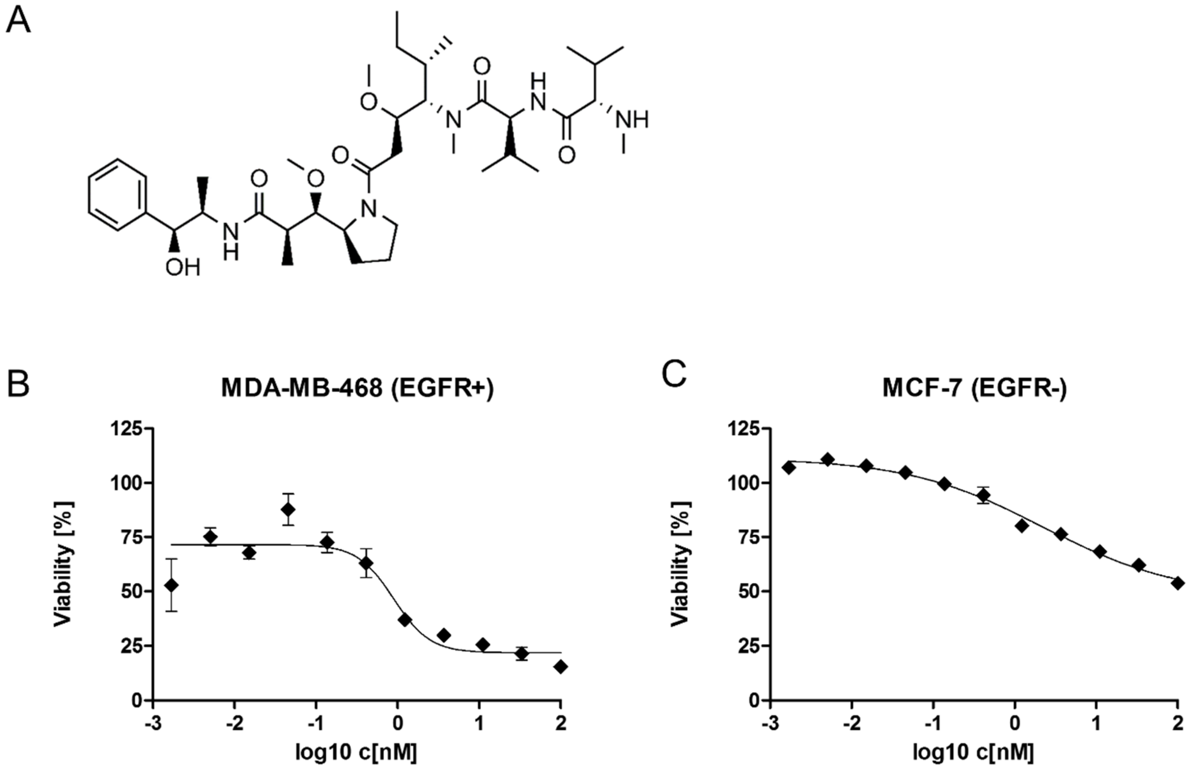

Figure S40. Cytotoxicity assay including free MMAE using the breast cancer cell lines MDA-MB-468 and MCF-7. (A) Structure of free MMAE. (B) *In vitro* cell killing of free MMAE using EGFR overexpressing MDA-MB-468 cells. (C) *In vitro* cell killing of free MMAE using EGFR-negative MCF-7 cells. Incubation times were 3 days. Error-bars were calculated from duplicates. MMAE was added in a serial dilution with the highest concentration of 100 nM.
